# Interoperability of health data using FHIR Mapping Language: transforming HL7 CDA to FHIR with reusable visual components

**DOI:** 10.3389/fdgth.2024.1480600

**Published:** 2024-12-19

**Authors:** Igor Bossenko, Rainer Randmaa, Gunnar Piho, Peeter Ross

**Affiliations:** ^1^ Department of Software Science, Tallinn University of Technology (TalTech), Tallinn, Estonia; ^2^ Department of Health Technologies, TalTech, Tallinn, Estonia; ^3^ Research Department, East Tallinn Central Hospital, Tallinn, Estonia

**Keywords:** FHIR Mapping Language (FML), TermX, semantic interoperability, data transformation, HL7 Clinical Document Architecture (CDA), HL7 Fast Healthcare Interoperability Resources (FHIR)

## Abstract

**Introduction:**

Ecosystem-centered healthcare innovations, such as digital health platforms, patient-centric records, and mobile health applications, depend on the semantic interoperability of health data. This ensures efficient, patient-focused healthcare delivery in a mobile world where citizens frequently travel for work and leisure. Beyond healthcare delivery, semantic interoperability is crucial for secondary health data use. This paper introduces a tool and techniques for achieving health data semantic interoperability, using reusable visual transformation components to create and validate transformation rules and maps, making them usable for domain experts with minimal technical skills.

**Methods:**

The tool and techniques for health data semantic interoperability have been developed and validated using Design Science, a common methodology for developing software artifacts, including tools and techniques.

**Results:**

Our tool and techniques are designed to facilitate the interoperability of Electronic Health Records (EHRs) by enabling the seamless unification of various health data formats in real time, without the need for extensive physical data migrations. These tools simplify complex health data transformations, allowing domain experts to specify and validate intricate data transformation rules and maps. The need for such a solution arises from the ongoing transition of the Estonian National Health Information System (ENHIS) from Clinical Document Architecture (CDA) to Fast Healthcare Interoperability Resources (FHIR), but it is general enough to be used for other data transformation needs, including the European Health Data Space (EHDS) ecosystem.

**Conclusion:**

The proposed tool and techniques simplify health data transformation by allowing domain experts to specify and validate the necessary data transformation rules and maps. Evaluation by ENHIS domain experts demonstrated the usability, effectiveness, and business value of the tool and techniques.

## 1 Introduction

Electronic Health Records (EHRs) are shared patient records that contain historical data about a patient compiled from all local Electronic Medical Records (EMR). EHRs serve a dual purpose in the healthcare ecosystem. Primarily, healthcare professionals use EHRs in healthcare delivery to access patient medical histories, diagnoses, treatments, and treatment outcomes (1). Additionally, routine clinical data is valuable for secondary use in clinical research, public health assurance, healthcare financing, and health policy-making (2) by enabling the aggregation and analysis of health data to improve healthcare (3, 4).

The European Health Data Space (EHDS) initiative (5) aims to build a health data sharing ecosystem (6) within the European Union (EU), establishing standards, practices, infrastructures and governance to support the primary and secondary use of EHRs (7). It facilitates healthcare access across borders in a mobile world where people travel for work and leisure (8). While the EHDS has ambitious targets to improve data sharing and patient access across the EU, there are concerns that it might be too large an undertaking to succeed (9). Additionally, it could undermine patients’ control over their data (10), complicate the work of healthcare professionals (9), and reduce public confidence (11). Furthermore, the challenges include inadequate compliance with existing regulations, such as the GDPR (12), potential excessive dominance and control by large tech companies (13), and deepening digital divides (14).

One possibility for adjusting the EHDS to more manageable goals with incremental steps is to utilize federated EHRs at different levels. These levels include the national level, such as the Estonian National Health Information System (ENHIS) (15), the healthcare institution level, such as in Austria where data is stored by the healthcare provider who first collected or generated it (16), and the citizen level, stored on citizens’ devices (17). A more radical federation approach involves decentralized content-addressable storage networks fully owned and controlled by citizens (18). Federated EHRs, particularly at the citizen level, offer several benefits compared to those stored in unified data silos (17, 18):

• *Privacy and security*: Reduces the risk of large-scale data breaches by allowing patient data to remain within national borders.
• *Single points of failure*: Reduces the risk of single points of failure, enhancing system resilience.
• *Patient trust*: Ensures transparency and control over data sharing, encouraging greater patient engagement in healthcare initiatives.
• *Compliance with regulations*: Supports compliance with national and EU regulations, particularly the GDPR, by keeping data within jurisdictions and providing patients with control over their health information.

Despite strong security and data protection properties, federated EHRs face a major challenge: semantic interoperability (19), which involves creating a common understanding of data elements and their relationships, aligning data structures, and standardizing terminology. Different healthcare providers often use different standards and vocabularies, leading to inconsistencies and data integration and interpretation difficulties. Even with the same standards and vocabulary, differences in interpretation arise (20, 21), whether among software developers or domain experts, including physicians.

### 1.1 Research problem

The article addresses the need for the semantic interoperability of health data in various formats. The ENHIS, operational since 2008 and maintaining lifelong health records of all Estonian citizens (15), is transitioning from the HL7 Clinical Document Architecture (CDA) format to Fast Healthcare Interoperability Resources (FHIR) (22). To mitigate the risks associated with data migration, the system must operate with legacy CDA data while storing new data in FHIR format, necessitating on-the-fly semantic interoperability between both formats.

In addressing the specific real-world issue of converting CDA to FHIR, we framed it as a broader problem of transforming EHR data from one format to another in a semantically interoperable manner.

### 1.2 Research questions

This paper focuses on using reusable components to transform health data from CDA to FHIR, an approach which serves as a methodical basis for developing and modernizing health information systems toward seamless semantic interoperability. It contributes to achieving federated semantic interoperability rather than integrated (common data format) or unified (common standard) interoperability (23). Federated interoperability allows different systems to work together coherently and efficiently, enabling dynamic networking with minimal costs (24). Each system can use its preferred data transmission protocol internally, with adapters performing the necessary conversions based on specified transformation rules and maps. Our paper provides tools and techniques for creating these transformation rules and maps, enabling semantic data transformations on the fly.

A Dutch study (25) compared CDA and FHIR representations for the inter-convertibility and consistency of Detailed Clinical Models (DCMs). While most aspects were adequately represented, issues with restrictions, coded values, narrative structures, and attribute meanings could lead to semantic challenges, emphasizing the need for the right DCM implementation standards. Austrian (26), Italian (27), and Estonian (28) studies demonstrate the potential for transforming International Patient Summaries (IPSs) (29) from HL7 CDA documents to FHIR resources. However, these transformations were hard-coded (30), making them opaque to business analysts, difficult to reuse, rigid, and challenging to maintain long-term (31).

Our goal is to provide a robust and reliable health data transformation process that can be replicated and reused in various contexts, with two important objectives:

• *The problem of clarity*: Implementing a low-code/no-code pattern should facilitate the faster delivery of transformations by minimizing hand-coding and utilizing a graphical user interface. Visual representation should conceal the complexity of the data transformation language, enabling analysts to adapt quickly. This strategy should increase efficiency and productivity and reduce dependency on developers.
• *The problem of reuse*: Reusing transformation rules and maps should save time and costs and improve efficiency, consistency, and readability. It should also lessen challenges such as initial investment, compatibility, and flexibility. Ensuring reusability requires careful planning and standardization. Visual representations can simplify understanding and apply complex transformations, while clear guidelines should facilitate reuse. This approach should enhance data processing quality and reduce the learning curve, fostering a more collaborative and efficient work environment.

Research rigor is centered on systematically developing visual mappings to facilitate data transformation. It emphasizes enhancing the clarity of transformations and promoting their reuse. This is demonstrated by customizing CDA and FHIR models, developing effective transformation rules and maps, and instantiating FML transformations.

### 1.3 Research results

Our work consolidates the experience of mapping and transforming data between HL7 CDA and HL7 FHIR R5 within the Estonian National Health Information System.

Using a Design Science (DS) methodology (32), we developed techniques for domain experts to create and reuse visual health data transformation components, along with preliminary techniques for ensuring their correctness.

After analyzing existing data transformation languages and tools, we support the use of the FHIR Mapping Language (FML). To address the lack of suitable tools for domain experts (33), we designed, developed, and validated the TermX tool (34, 35) with input from domain experts (36, 37). TermX allows domain experts to specify and test transformation rules and maps between data formats using a WYSIWYG^1^ approach with minimal technical knowledge (38).

### 1.4 Outline of the paper

The paper is organized as follows: Section 2. explains the HL7 CDA to FHIR transformation challenges, the TermX tool we developed for data transformations, and the methods we use in creating the data transformation techniques. Section 3. documents the transformation techniques. Section 4. evaluates the proposed techniques and discusses the related social impacts in the context of the EHDS. It also discusses related work, including an analysis of the pertinent tools and languages. Finally, in Section 5, we conclude and outline directions for future research.

## 2 Methods

We aim to improve data transformations by designing techniques and reusable WYSIWYG transformation components that domain experts can use to specify and validate data transformation rules and maps for semantic interoperability in EHR infrastructure, with only minimal technical expertise and skill needed. We adhere to the Design Science (DS) methodology (32, 39). A *transformation rule* is a specific instruction or set of instructions that defines how a particular piece of data should be transformed (40). A *transformation* refers to the overall process of converting data from one format or structure to another (40). A *transformation map* is a set of transformation rules and metadata used by the transformation engine during the transformation process (41). A *transformation component* is a visual representation of a transformation rule or map in TermX Visual Editor that contains an FML code that makes the necessary transformations. The techniques and transformation components, along with the TermX tool we use, are our artifacts. The context of these artifacts in performing health data transformations is the IT infrastructure of health organizations and state agencies. DS problems are improvement problems. This work aims to improve the federated semantic interoperability between heterogeneous healthcare EHRs. The proposed techniques are illustrated with data transformations from CDA to FHIR.

**Figure 1:**
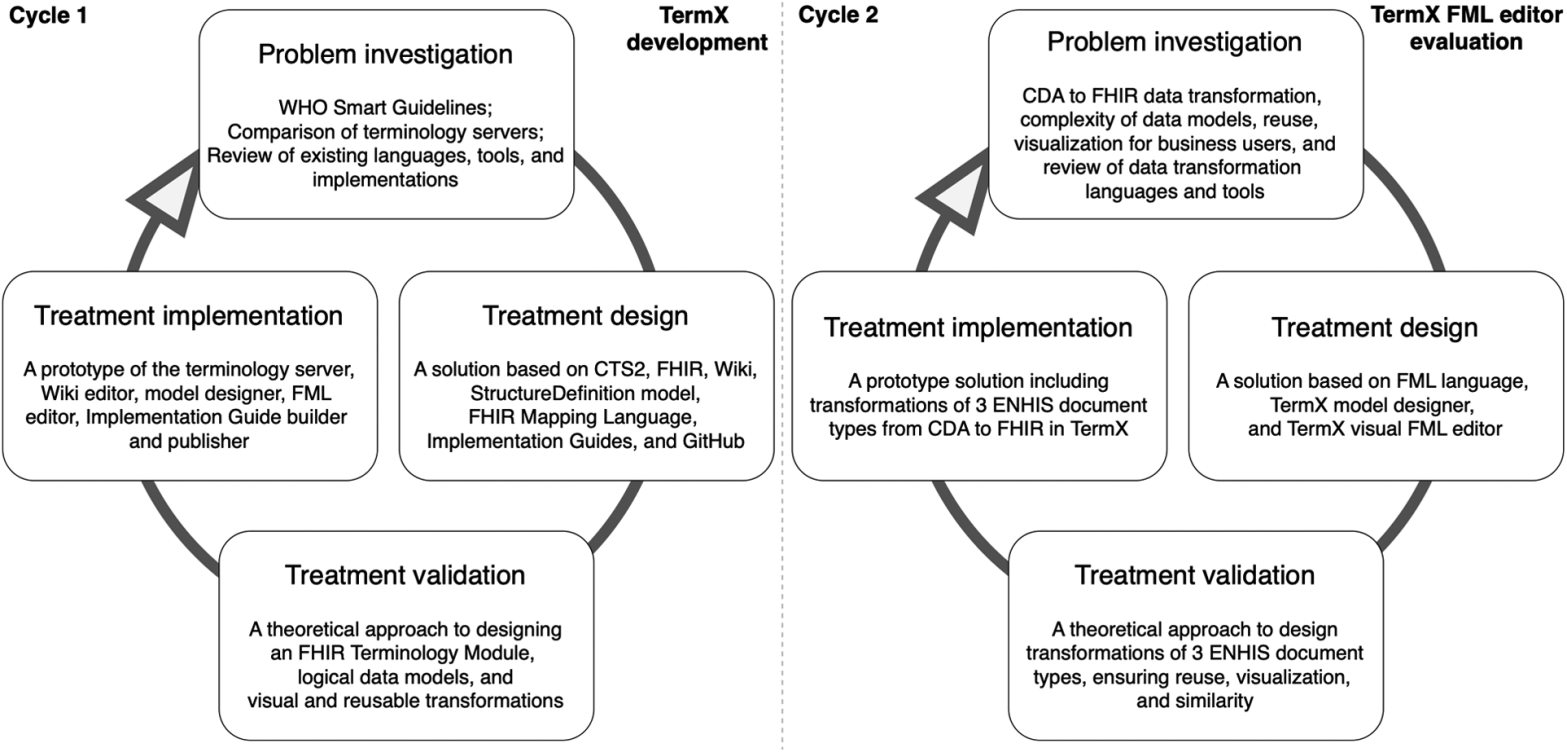
The Design Science methodology used for the development and evaluation of TermX.

DS is part of the engineering cycle (Figure 1) and includes the problem investigation, treatment design, and treatment validation phases. The treatment implementation phase is not part of DS but forms an engineering cycle along with the DS phases. This paper reports two DS cycles and therefore also two engineering cycles. In the first cycle, we designed and developed the TermX tool. In the second cycle, we evaluated the TermX tool by designing the techniques and reusable WYSIWYG components for data transformation rules and maps from CDA to FHIR.

While the implementation of the artifact (TermX tool) is not part of DS but part of the engineering cycle, Figure 1 includes its implementation to illustrate the place and role of the TermX tool’s development in our study. We designed TermX according to the DS methodology, encompassing the following steps: (1) investigating a problem, problem relevance, and research rigor by reviewing published papers on existing data transformation languages, tools, and implemented projects (see Section 4.1); (2) designing the TermX tool (38); and (3) validating the TermX design with domain experts from various countries (see Section 2.2).

In the second cycle, the main focus of the current paper is to evaluate the TermX tool by designing visual reusable transformation components that domain experts can use for CDA to FHIR transformations. We also generalize the transformation components’ development process as techniques for developing reusable transformation components using TermX (Section 3) and explain the relevance of our research in the EHDS ecosystem, including how the proposed approach supports federated semantic interoperability (Section 4).

### 2.1 HL7 CDA to FHIR transformation

HL7 CDA (42) and HL7 FHIR (43) are two widespread standards for the interoperability of health information systems. Although these two standards are designed to be interoperable, the semantic heterogeneity of various software vendors’ implementations inhibits semantically correct model transformations between these standards (44). Additionally, model transformations between specific HL7 CDA and HL7 FHIR implementations are not straightforward and there is no single correct way to achieve them (27). Therefore, highlighting a new tool and the related techniques is pertinent, as transformation techniques between CDA and FHIR are relatively undocumented in academic literature.

HL7 CDA is a template-based and XML-centric standard for health data documents, first released in the early 2000s (42). It is a complex standard with many shortcomings in data redundancy and analysis. HL7 FHIR, by contrast, is a modern interoperability framework based on widespread web technologies, such as REST and JSON (44, 45). The shortcomings of HL7 CDA have been largely addressed in FHIR, which is why mapping and transforming existing HL7 CDA formatted health data to HL7 FHIR resources in a semantically interoperable way has tremendous potential and value in both health data usage and health data analysis-related innovation (46).

Although CDA and FHIR are designed to be interoperable, both standards are complex, and transformation between them is non-trivial (46). For example, the HL7 Reference Implementation Model (RIM) used within HL7 V3 and CDA aims to encompass the full spectrum of possible healthcare scenarios (47). In contrast, HL7 FHIR provides a model for the most common scenarios. Instead of defining a complete model for all aspects of healthcare, FHIR follows the 80/20 principle by defining only the most common health scenarios, adding the possibility of extension to cases where customization is necessary (48, 49).

The FHIR authors have identified various interoperability challenges when transforming data from CDA format to FHIR. Key points include clinical content mapping at the template level, managing differences in narrative granularity, and handling discrete-to-human-readable linkages, with some potential information loss when converting from CDA to FHIR (50). Additionally, both CDA and FHIR standards have evolved over time, and each new version brings changes that may not be compatible with previous versions (51–53). Efforts also exist to maintain forward and backward compatibility between versions, which is not guaranteed in all cases (53).

It is important to note that while CDA and FHIR are specifications for health data exchange, they differ in their approach and usage. FHIR’s resource-based model allows for more granular control and flexibility, whereas CDA’s document-centric approach provides a robust and standardized format for clinical documents. They also differ in their licensing requirements: CDA requires a license for use, whereas FHIR is dedicated to the public domain to encourage widespread adoption.

### 2.2 TermX: a game changer in interoperability

**Figure 2:**
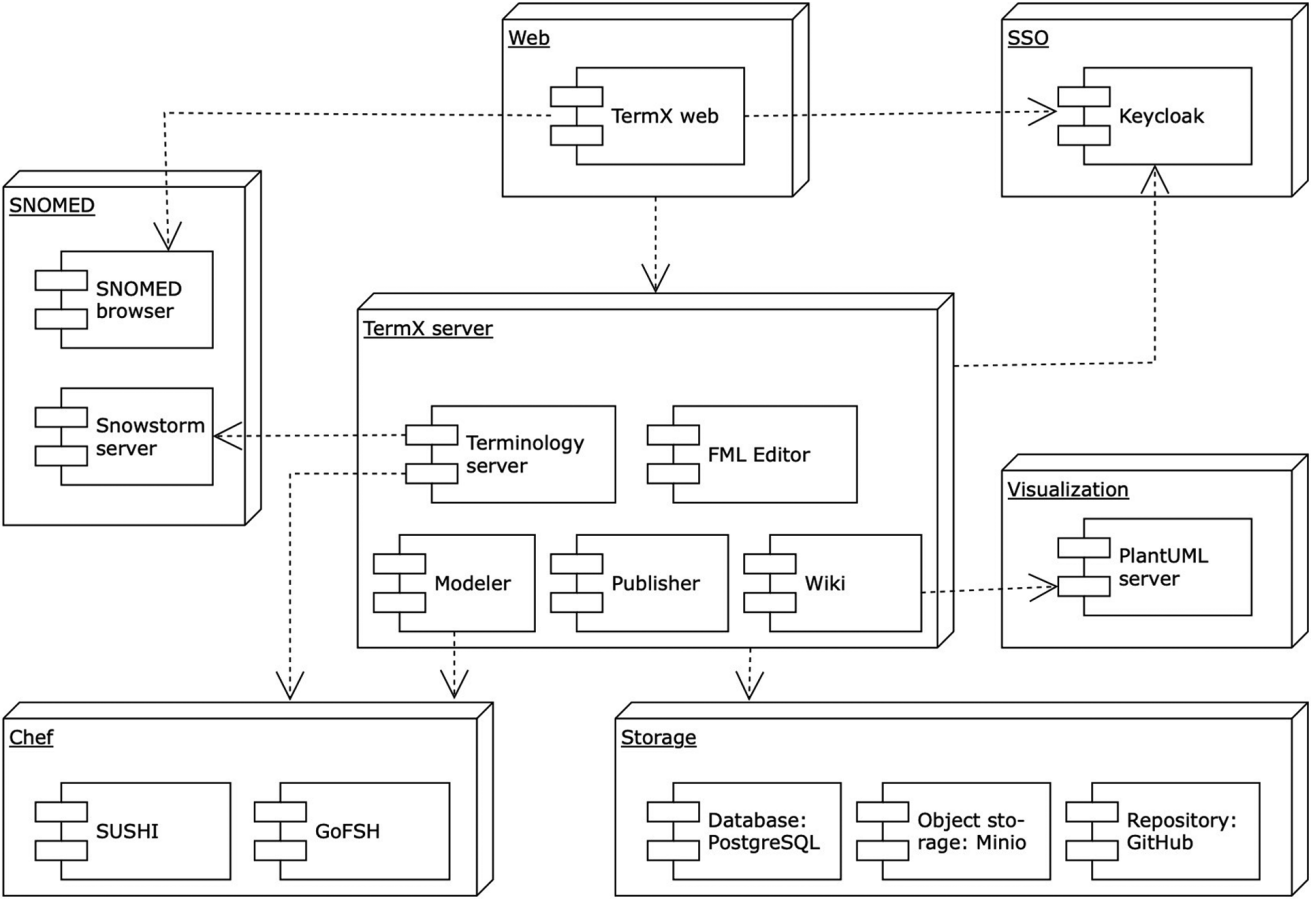
TermX component diagram.

The necessity of robust, enduring, and relevant healthcare interoperability is universal across all clinical and health domains. However, we identified a gap in the availability of open-source, cost-free, high-quality tools that offer multilingual support and an advanced graphical interface (33). To address this, we designed and implemented TermX – a novel, open-source platform for terminology management and data transformations to support interoperability between healthcare institutions and systems (34). TermX incorporates a terminology server, a Wiki, a model designer, an FML transformation editor, and tools for authoring and publishing (35). Figure 2 visualizes the TermX components (38). TermX is designed to manage data models and transformations and develop terminology and implementation guides for healthcare systems at international, national, regional, and hospital levels. It aims to ensure open, standardized access to published data and guarantee semantic interoperability based on the FHIR standard. We have validated TermX with TalTech (Tallinn University of Technology, Estonia), the private sector, and national standardization agencies in Estonia, Lithuania, Uzbekistan, and the Czech Republic.

**Figure 3:**
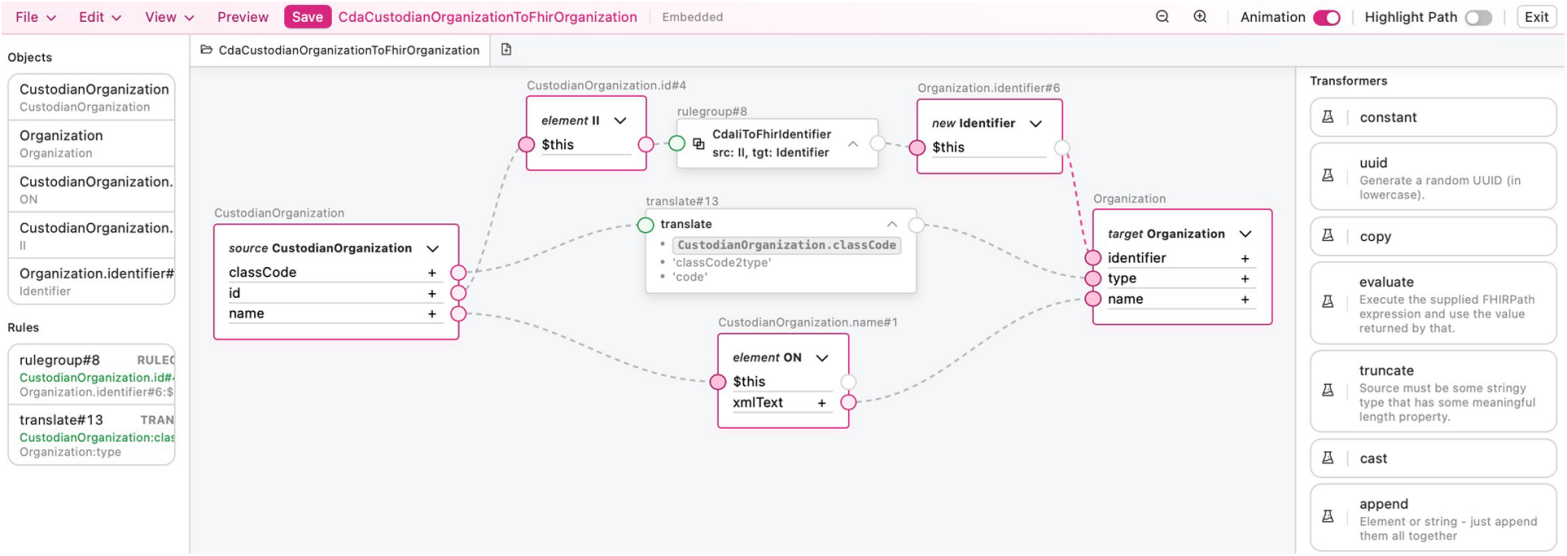
User interface of the TermX FML editor.

TermX provides a visual model designer and FML Editor for creating and visualizing data models and FML transformation rules and maps through a user-friendly interface (Figure 3). They are designed specifically for business analysts rather than developers. The model designer implements the FHIR StructureDefinition specification (54) and provides the capability to manage data models through a user-friendly interface or formal specification in FML code. The FML editor’s core purpose is to design transformation components, hide the complexity of the CDA, FHIR, and FML languages, and enable analysts to adapt quickly.

TermX uses the FHIREST (55) and HAPI FHIR (56) libraries to provide the FHIR API and uses HAPI FHIR (57) as the foundation for its transformation engine, transforming data from input sources into output sources (38). TermX was created as the result of an academic project at TalTech.

#### 2.2.1 Reusable visual transformation components

**Figure 4:**
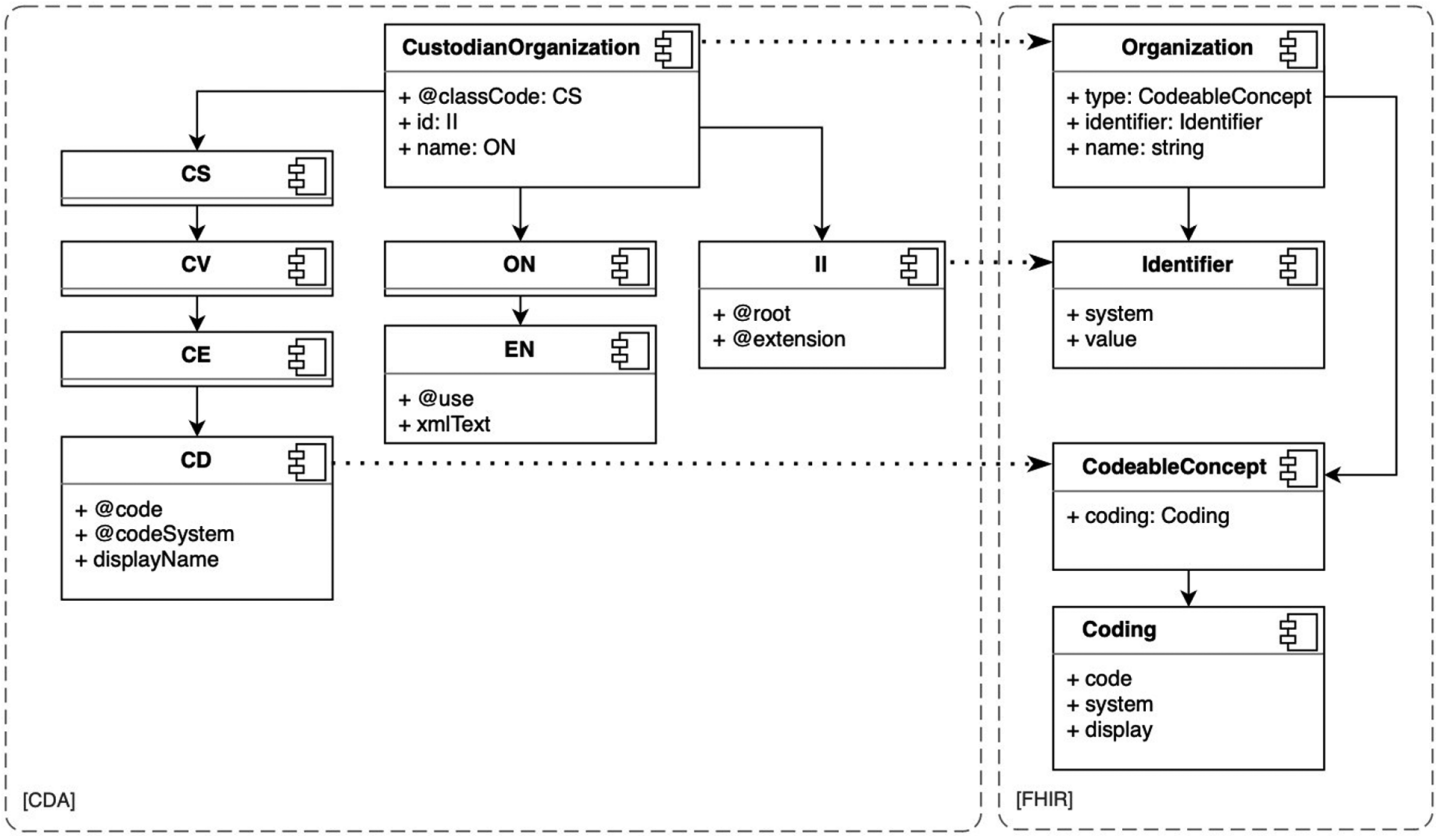
CDA CustodianOrganization and FHIR Organization resources, subtypes and related mappings.

CDA and FHIR are health data interoperability models developed by HL7 (44); both are designed with a hierarchical structure of data types and resources. For instance, CDA includes four code data types: CS (code simple), CV (coded value), CE (code with equivalents), and CD (concept descriptor) (see Figure 4). CS is the simplest, while CD is the most complex. Complex data types are composed of simple data types. In CDA, the simplest data type may be a subset of a more complex data type, for example, a CS is a subset of a CV data type. In FHIR, resources are categorized into metadata, special-purpose, general-purpose, and primitive data types (58). In both models, the depth of objects in the XML or JSON document tree can become very large. In the case of large CDA documents, the depth of the document trees results in very voluminous transformations.

The transformation is the entire process of converting the resource, while the transformation rules are the detailed steps that specify how each attribute within the resource should be handled. Transformation rules are applied to convert the extracted data from its previous form into the required form. These rules could involve various instructions, such as extraction, conversion, or formatting. The transformation map, conversely, is not just an abstract concept but manifests itself as a tangible artifact. Every transformation map may be reused as a transformation rule in another transformation. Correct transformation rules and maps are fundamental in defining transformations, such as transforming CDA documents to the FHIR Bundle resource (59), as needed in the ENHIS. We identified the required transformation rules and maps between the data types and models of these two standards and created corresponding transformation components. We found that transformation components of simple data types, such as CD to CodeableConcept and II (instance identifier) to Identifier (see Figure 4), can be reused in more complex data types and model transformations. Such reuse simplifies the development of transformation rules and maps, improves clarity, and reduces the needed FML source code.

### 2.3 Research towards reusable visual transformation techniques

#### 2.3.1 Problem investigation

The data transformation from CDA to FHIR necessitates a profound comprehension of the data structures inherent in both standards. FHIR *StructureDefinition* (54) describes a resource structure and defines a set of data element definitions and associated usage rules. These structure definitions describe the content defined in the FHIR specification, such as resources, data types, and underlying infrastructural types, and how these structures are utilized in implementations.

In CDA, each element is comprehensively defined using standard schema definition (XSD) files. These XSD files act as architectural designs, delineating the structure and data types of CDA documents and simplifying the process of validating these documents against the prescribed schema. The CDA model is based on the HL7 Reference Information Model (RIM) and utilizes reusable data types, templates, sections, and components (50). For instance, patient demographics, medication information, and clinical observations are standardized and reused across different CDA documents. HL7 has implemented a representation of the CDA R2.0 specification using FHIR Logical Models expressed as FHIR StructureDefinition instances available under an open-source license (60).

Many models in CDA and FHIR have numerous attributes, are complex, and contain hierarchies. We need a way to reuse data type transformations and provide reusable transformation components for CDA and FHIR subtypes, such as CD to Coding and II to Identifier. This approach will enhance the efficiency and reliability of data-handling processes. For instance, the ENHIS “Outpatient Case Summary” comprises 24 sections, while the “Birth Summary” comprises 17 sections (61). Of the “Birth Summary” sections, only four are absent in the “Outpatient Case Summary”. Our techniques involve creating transformation components for a single document type and then applying these components to different types of documents. If new sections are introduced in the new document type, transformation components are only developed for these new sections and included in the reusable transformation components library. With each new document type, the number of sections requiring transformation components development will decrease and eventually reach zero. We also need a solution to validate transformation components to identify problems during development rather than production and to avoid errors during the development of transformation components.

Transformations of simple data objects are straightforward, and the associated source code in FHIR Mapping Language is relatively uncomplicated. However, with the transformation of hierarchical complex objects, the source code becomes highly intricate and may pose comprehension challenges for domain experts. Complex transformations necessitate visualization (62). We aim to establish a set of CDA and FHIR transformation components encompassing a broad spectrum, ranging from primitive data types to complex resources, and formulate appropriate techniques. We hypothesize the following:

(1) TermX as an artifact will apply to all CDA data types, sections, and documents.
(2) All transformation components can be developed using the TermX visual user interface.
(3) The developed transformation components can be reused.

This strategy would facilitate the reuse of prior transformation components, thereby augmenting the efficiency and uniformity of transformation procedures. Such an approach is designed to fortify the robustness and adaptability of the developed TermX tool, equipping it with the capacity to help domain experts develop and validate transformation components by hiding the details and complexities embedded within CDA and FHIR data models.

#### 2.3.2 Treatment design

Based on the problem investigation above, we have established the following requirements for the visual reusable transformation components set:

(1) It must support strict data models
(2) It must support the reuse of transformation components
(3) It must have native support for CDA and FHIR
(4) It must support the WYSIWYG approach

This approach underscores our commitment to advancing the field of data transformation and management, ensuring that our data transformation techniques are accessible and understandable to a broad range of stakeholders.

The selected approach evaluates the usability of the TermX model designer and the TermX visual FML editor, the FML language, and the HAPI FHIR implementation of FML used by TermX (Figure 2 illustrates the TermX architecture and components). TermX enables the registration of HL7 V3 and CDA models in the TermX model designer, uses FHIR resource definitions, creates data transformation rules from CDA to FHIR in the TermX visual FML editor, and publishes the transformations on GitHub.

The transformation may be triggered by HTTP requests within scripts or through the web user interface. TermX is available as a set of Docker containers used for deployment. We use the logical models provided with the HL7 CDA R2.0 core standard (60) as a basis for ENHIS CDA input instances. These models were extended according to the ENHIS CDA standard implementation. We used FHIR Release 5 (R5) structure definitions (54) as the standard for output instances. The transformations handle one input CDA file and output one FHIR file.

#### 2.3.3 Treatment validation

Treatment validation ensures that the chosen approach contributes to achieving stakeholders’ goals when implemented. Our approach includes prototyping a set of transformation components using ENHIS version 8.2 CDA documents, the FHIR R5 specification, and the TermX tool. The FML Editor achieved Technology Readiness Level (TRL) 5 according to the European Commission’s classification (63) at the start of the validation process. The dataset, derived from three ENHIS HL7 CDA document types: the “Outpatient Case Summary”, the “Notice of Growth”, and the “Birth Summary”, was established during the research to validate the proposed transformation techniques. For each selected document type, we used a sample CDA document from the ENHIS specification that includes all available sections.

The ENHIS “Outpatient Case Summary” includes 24 data sections, the “Notice of Growth” includes seven sections with two unique sections, and the “Birth Summary” includes 17 sections with four unique sections. Initially, we developed transformation components for all sections in the “Notice of Growth” and their associated classes and data types. Additionally, we created a transformation component to convert the “Notice of Growth” document into FHIR, incorporating all the transformations in the created section. For each subsequent document, we created a new transformation component that included the transformation components of the existing sections. Then, we added new section transformation components and linked them to the particular document transformation component. With the implemented prototype, we successfully verified that: (1) TermX was applicable for all necessary CDA data types, sections, and documents; (2) all transformation components were developed using the TermX visual user interface; and (3) the developed transformation components were reused in subsequent data types, sections, and documents.

The results obtained were first validated manually by comparing CDA and FHIR messages section by section to ensure the correctness of transformations. Next, we designed a technique (Section 2.3.4) to automate the validation process. Subsequently, the results were demonstrated to the IT department of the Health and Welfare Information Systems Centre (TEHIK), which operates the ENHIS. The feedback was overwhelmingly positive, with the team expressing their approval and satisfaction. Following the internal evaluation, TEHIK chose it as their transformation tool.

#### 2.3.4 Advance techniques for validating transformation rules

Transformation validation should be deterministic, with each transformation having a dedicated test suite using predefined human-validated inputs and expected outputs. While developing these deterministic input-output pairs is time-consuming and can lengthen the development cycle, it is essential for robust production solutions and sometimes required by legislation (64, 65). We envision quicker heuristic feedback techniques for prototyping or experimentation, combining FHIR structure validation and an input-output content similarity assessment using a natural language processing (NLP) solution. However, supporting dedicated test suites in TermX and developing these heuristic validation techniques will largely be a part of future work.

Data similarity between the original HL7 CDA and the transformed HL7 FHIR documents was validated. No specialized out-of-the-box tool capable of statistically evaluating the correctness of the transformations was found. Therefore, CDA and FHIR documents were converted into collections of key-value pairs to which statistical tools were applied (66). The highest similarity percentage was achieved using the Term Frequency-Inverse Document Frequency (TF-IDF) methods (67). Further research in this direction is planned for the future.

## 3 Development techniques for reusable visual transformation components

Our study results in developing hierarchical, reusable transformation components for converting CDA documents into the collections of FHIR resources [Bundle (59)]. It highlights techniques that use the FHIR Mapping Language and the TermX visual editor to improve reuse and clarity in data transformations. First, we introduce the devised techniques. Then, we illustrate how the visual TermX editor supports our approach, making it accessible to analysts through a no-code visual interface. We provide practical examples using the ENHIS CDA documents, specifically the “Notice of Growth”, “Outpatient Case Summary”, and “Birth Summary”, to demonstrate the application of these techniques in real-world scenarios. Furthermore, we outline preliminary techniques for validating transformation components, emphasizing the need for deterministic testing and proposing heuristic feedback techniques.

### 3.1 Techniques for hierarchical reusable transformation components

**Figure 5:**
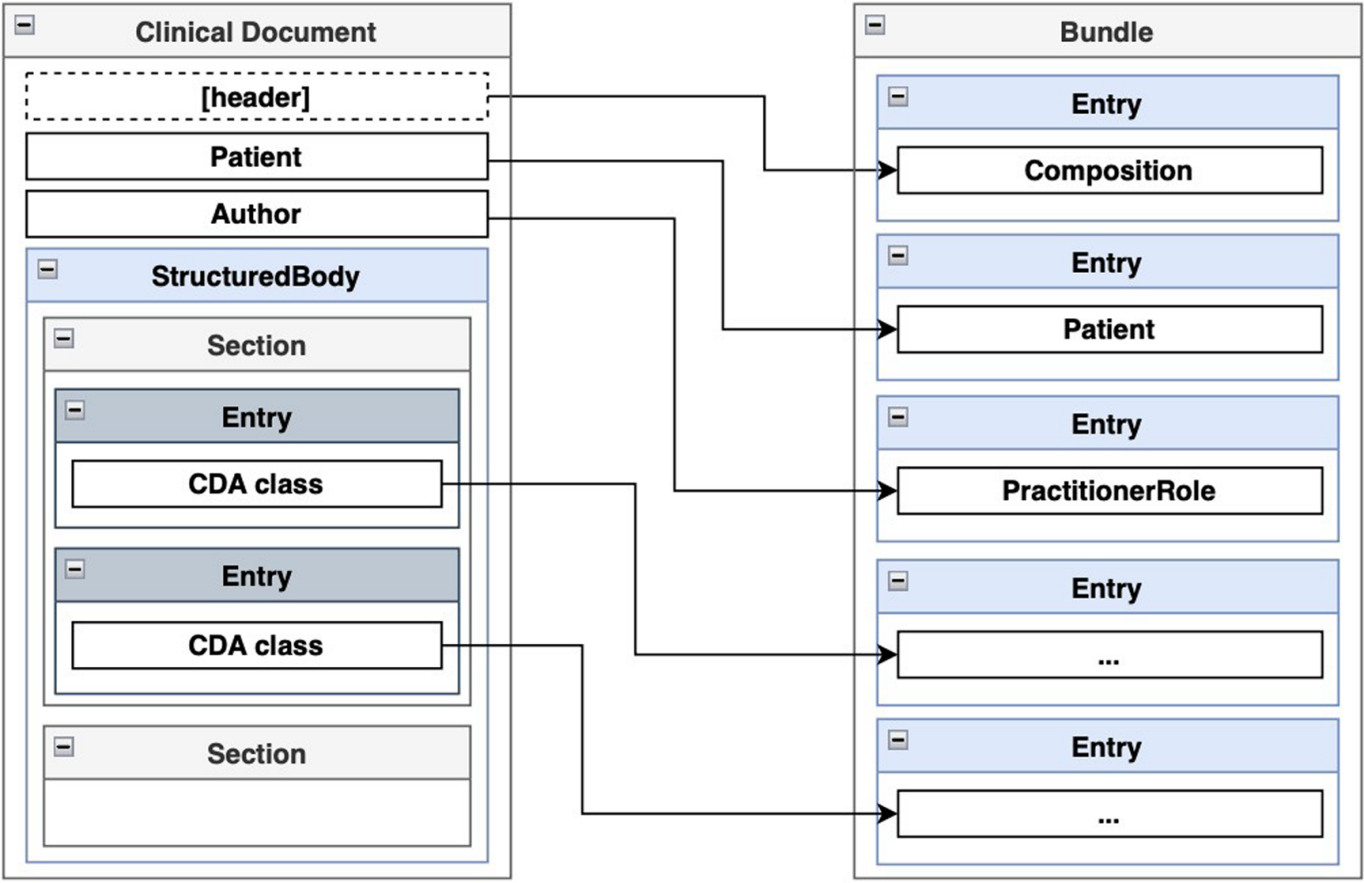
Mapping of ClinicalDocument to FHIR Bundle.

According to the authors of FHIR, transformations from CDA to FHIR should be performed at the template level (50). A CDA template follows a specific structure: the entire document is encapsulated within a <*ClinicalDocument*> element, which includes header information and a <*structuredBody*> element. The <*structuredBody*> element is composed of <*component*> elements, which in turn consist of <*section*> elements (Figure 5). These <*section*> elements comprise standard HL7 CDA classes, with optional extensions defined by the implementer. CDA classes are assembled using other CDA classes and complex and primitive data types. FHIR resource definitions also use other definitions and data types. A transformed CDA document is presented as an FHIR Bundle—a container holding a collection of FHIR resources.

We propose that the issues of reuse and clarity in CDA to FHIR transformations can be addressed through a hierarchy of reusable transformation components organized similarly to the structure of a CDA document. The FHIR Mapping Language allows the reuse of transformation maps that can be invoked from other transformation rules, thereby supporting our proposed approach.

We commence by delineating a hierarchical structure of data types and models. This hierarchy is instrumental in encapsulating the complexity and diversity of healthcare data. The fundamental units can be categorized into primitive, basic, and complex data types. Each of these categories represents a different level of abstraction and complexity. Primitive data types are the simplest and most fundamental, representing basic data elements such as strings and numbers. Basic data types are slightly more complex, encapsulating the related data elements. Complex data types, on the other hand, represent a collection of basic and primitive data types, forming a more intricate structure. Subsequently, we establish transformation components between these data types. These transformation components elucidate the relationships and transformations between data types, thereby facilitating interoperability and data exchange. Lastly, we construct transformation components between different models.

Our findings demonstrate that it is feasible to define reusable transformation components at various levels of granularity of a CDA template: the complex data type level, the CDA class level, the section level, and the document level. The primitive data types between CDA and FHIR are already interoperable. Based on these levels of granularity, we establish sets of transformation rules to be maintained.

**Figure 6:**
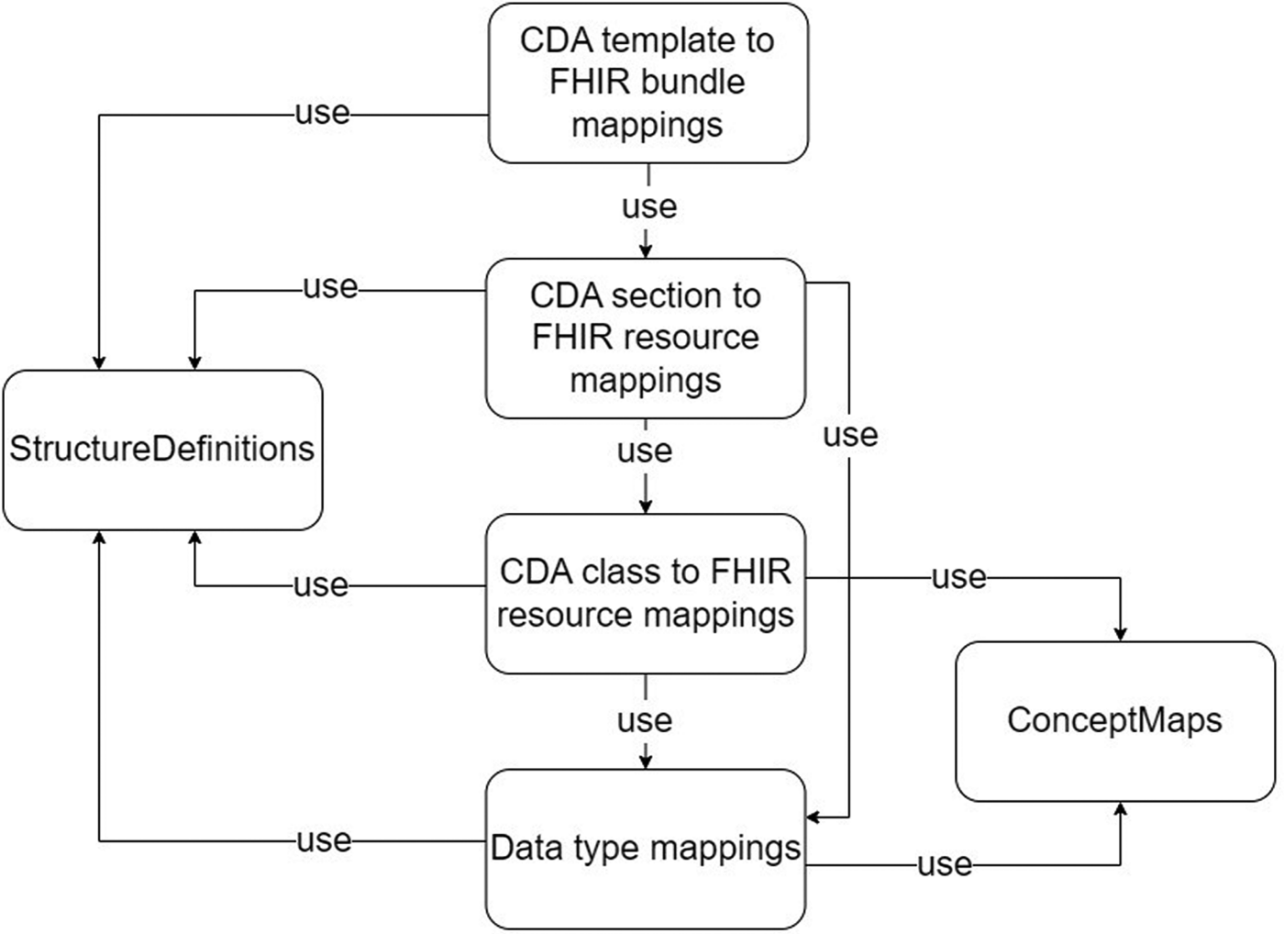
Usage dependencies between artifacts used in CDA to FHIR mappings.

With the different granularity level transformation components, a set of *ConceptMap*, and the source and target *StructureDefinitions*, we define a set of software artifacts to be created and maintained for developing robust CDA to FHIR transformation components quickly. The list of artifacts is described in Table 1, and the dependencies among the artifacts are visualized in Figure 6. We designed the transformation components to transform basic and complex data types from CDA to FHIR. Mappings from CDA sections to FHIR resources are assembled using CDA class to FHIR resource transformation components and CDA complex data type to FHIR complex data type transformation components. Subsequently, the CDA document for FHIR bundle transformation components can be formed using the CDA section for FHIR resource transformation components. The CDA document header is considered a section in our approach. Lower levels of granularity transformation components are used in the transformation components with the higher granularity level, thus adhering to one-way dependencies—an important software architecture pattern.

**Table 1:** CDA2FHIR artifacts.

Artifact	Source	Target	Explanation
I/O structures			The definitions of the structures for the inputs and outputs of the transformations in the form of FHIR StructureDefinition resources.
Classifier mappings			FHIR ConceptMap resources that map CDA coding systems to FHIR coding systems.
Data type Mappings	CDA data type	FHIR data type	Transformations between CDA data types and FHIR data types in the form of FML or FHIR StructureMap resources.
Class to Resources Mappings	CDA class	FHIR resources	Transformations between CDA classes and FHIR resources in the form of FML or FHIR StructureMap resources, constructed from the elements of data type transformations and classifier transformations.
Section to Resources Mappings	CDA <*section*>	FHIR resources	Transformations between CDA document sections and FHIR Bundle resources in the form of FML or FHIR StructureMap resources. A document section is a code-distinguished section within the structuredBody element of a CDA document or the CDA document header. These transformations are constructed from the elements of transformations between CDA classes and FHIR resources as well as data type transformations.
Document to Bundle Mappings	CDA document template	FHIR bundle	Transformations between CDA documents and FHIR Bundle resources in the form of FML or FHIR StructureMap resources. These transformations are constructed from the elements of transformations between CDA document sections and FHIR Bundle resources.

In addition to these transformation components, two additional components are required. The *ConceptMap* (68) translates the set of concepts in one code system to one or more concepts in other code systems. The *StructureDefinitions* (54) are used to define source and target data models of the transformations.

The reuse problem is addressed using a single transformation component in multiple other transformation components where the same construct is mapped. For example, a component that maps a CDA *II* class to a FHIR *Identifier* data type can be used in components mapping both the CDA class *CustodianOrganization* to the FHIR *Organization* resource and the CDA class *AssignedAuthor* to the FHIR *Practitioner* resource. By solving the problem of reuse, we ensure that issues in transformations have a single point of failure, thereby enhancing the robustness of the transformations. Reuse also enables the faster development of transformation components from CDA templates to FHIR bundles, as it eliminates the need to repeatedly write the same transformation component for transforming the same section or class to FHIR when working with different CDA templates.

The problem of clarity is addressed through reusable transformation components that encapsulate complexity at various levels of granularity. When analyzing a component that transforms a CDA template to an FHIR bundle using our proposed techniques, we only need to understand the different sections defined in the template without being burdened by the details of the transformation component of CDA classes or complex data types. This principle applies to rules at each level of granularity, ensuring that each component remains focused and comprehensible by abstracting lower-level details.

### 3.2 Techniques for visualizing transformation components with TermX

To support the described techniques for developing CDA to FHIR transformation components using FML, a visual editor must support the following use cases: the management of *StructureDefinitions*, the management of *ConceptMaps*, the creation of FML transformation, and the ability to use existing transformation components in other FML transformations. According to our results, the TermX software supports all of these use cases through a visual user interface with low-code/no-code.

In TermX, the management of *StructureDefinitions* is part of the Modeler module. *StructureDefinitions* can be displayed as a tree-like visual structure and edited without modifying the underlying JSON or FHIR Shorthand (FSH) (69) source. Additionally, the HL7 CDA *StructureDefinitions* do not need to be implemented from scratch, as the FHIR authors have provided multiple core standard CDA specifications using FHIR Logical Models expressed as FHIR *StructureDefinition* instances (60). These logical models can serve as a basis for *StructureDefinitions* of a specific CDA implementation. The CDA *StructureDefinitions* can be created in TermX using the provided JSON or FSH syntax and then edited with the visual editor to fit specific implementation guidelines. A FHIR implementation generally includes an *Implementation Guide* containing the Resources’ *StructureDefinitions*.

The Terminology module supports the management of *ConceptMaps* that represent the mapping between source and target terminology. The *ConceptMaps* can be used as a *transformation rule*.

TermX provides a visual FML editor as a designer of explicitly designed FML transformation components for business analysts (38). Every transformation has at least one source and target *StructureDefinition* and may reuse other FML transformation components and *ConceptMaps*. The imported elements can then be utilized on a visual canvas, dragging and dropping as boxes. Lines can be drawn between the boxes, visually modeling the control flow of the transformation rule from the source structure to the target structure, from which FML code is generated (Figure 3). The objective of the FML editor is to visually represent transformation rules, hide the complexity of the FML language, and facilitate rapid adaptation to the FML language.

In the work described in this paper, all the necessary transformation components were created with the visual editor of TermX; even the code generated behind certain transformation component visualization boxes and lines was not always intuitive to inexperienced users.

### 3.3 Techniques for developing CDA to FHIR transformation components

We evaluated the viability of the proposed techniques by developing a prototype development for transforming the ENHIS CDA documents “Notice of Growth”, “Outpatient Case Summary”, and “Birth Summary”. We began by dividing the “Notice of Growth” into sections and then breaking those sections into classes and data types. We also documented the necessary *ConceptMaps* and *StructureDefinitions*. After this, we developed the transformation components, starting with lower granularity artifacts. This process was repeated for the other two CDA documents, reusing already specified transformation components wherever possible. Subsequently, we provide examples from a real-world use case to illustrate the key points previously highlighted.

#### 3.3.1 Specifying CDA data type level transformations

For the ENHIS CDA *StructureDefinitions*, we were able to use the logical models provided with the HL7 CDA R2.0 core standard (60) as a basis, which were then modified as needed according to the ENHIS CDA standard implementation. This implementation is available as Enterprise Architect models and PDF documents on the web and is accessible within the Estonian IP address space. The modifications required for the core standard *StructureDefinitions* were necessary to address the extensions of the base model defined in the Estonian implementation as well as instances of misuse of the standard. For example, in the CDA *Observation* class, the *Ratio* data type for the value attribute is denoted as *RTO-PQ-PQ* in the core standard, which employs hyphens. However, in the ENHIS implementation, it is referred to as *RTO_PQ_PQ*, where underscores are used instead. An example of an extension that needed to be accounted for is the <*asLicencedEntity*> element added to the <*assignedEntity*> element to provide information about the authority licensing the healthcare worker. As the transformation target structure, we used the base FHIR R5 release, for which we utilized URIs in a test server.

**Figure 7:**
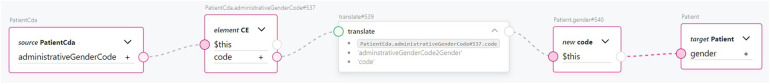
Transformation of CDA administrative gender attribute to FHIR gender attribute using ConceptMap for concept translation.

An example of using *ConceptMaps* and terminology translation between CDA and FHIR is illustrated when transforming the CDA Patient class into the FHIR Patient resource. The two standards use different sets of codes to represent the administrative gender of the patient. For instance, in the ENHIS CDA implementation, the code “N” represents the female gender, whereas in FHIR R5, the code “female” is expected. A *ConceptMap* was constructed and used with the transformation rule to perform translation between the two terminology code systems, as shown in Figure 7. In the figure, the *administrativeGenderCode* attribute of the Patient CDA class is piped into the transformation rule, the result of which is assigned to a new FHIR code data type and then to the gender attribute of the Patient FHIR resource.

**Figure 8:**
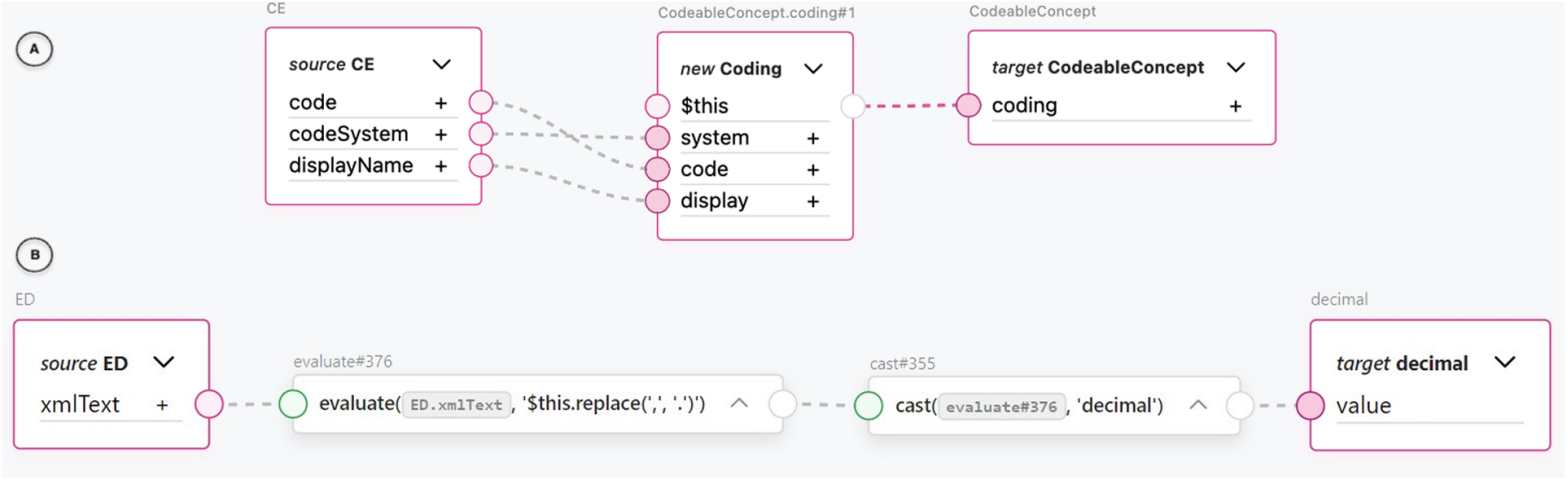
An example transformation from CDA CE class to FHIR CodeableConcept resource.

One of the most common transformations we encountered was between the FHIR concept and different representations of the CDA concepts. For example, FML transformation rules between the CDA CD class and the FHIR *CodeableConcept* resource as well as between the CDA CE class and the FHIR *CodeableConcept* resource provided significant value in terms of reuse. These transformation rules were very common in higher granularity level transformations. Due to the nested structure of the FHIR *CodeableConcept* and the three data attributes mapped between the structures, calling a reusable transformation rule with one line of code saved us from repeating the same six lines of code each time. An example of a reusable CDA CE to FHIR *CodeableConcept* transformation rule using the TermX visual editor can be seen in part A of Figure 8. The attributes of the CE CDA class are assigned to a new Coding FHIR resource. The Coding resource is then assigned to the target *CodeableConcept* coding attribute. Specifically, the CE CDA class’s code attribute corresponds to the FHIR Coding’s code attribute, the *codeSystem* attribute corresponds to the *system* attribute, and the *displayName* attribute corresponds to the *display* attribute.

Notably, FML also enabled us to handle semantically faulty XML at the data type level. In an *Observation* element in the “Outpatient Summary” test documents we used, we encountered a decimal value represented as text with a comma decimal separator inside an *EncapsulatedData* data type: <*value xsi:type=“ED”*>12,2</*value*>. To fix this issue, we were able to replace the decimal separator and cast the text into a decimal data type using FML’s *evaluate* rule with a *FHIRPath* expression and a *cast* rule. We accomplished all of this using only the visual editor (see Figure 8 part B). The inner text of the XML tag represented by the *xmlText* attribute is piped into an *evaluate* block, where a *FHIRPath* expression is used to replace the comma with a period in the text string. The evaluated string is piped into a *cast* block, which casts it to a decimal data type and assigns it to an output value. In our opinion, this result illustrates that a visual editor can produce fault-tolerant and robust transformation rules.

#### 3.3.2 Specifying CDA class level transformations

**Figure 9:**
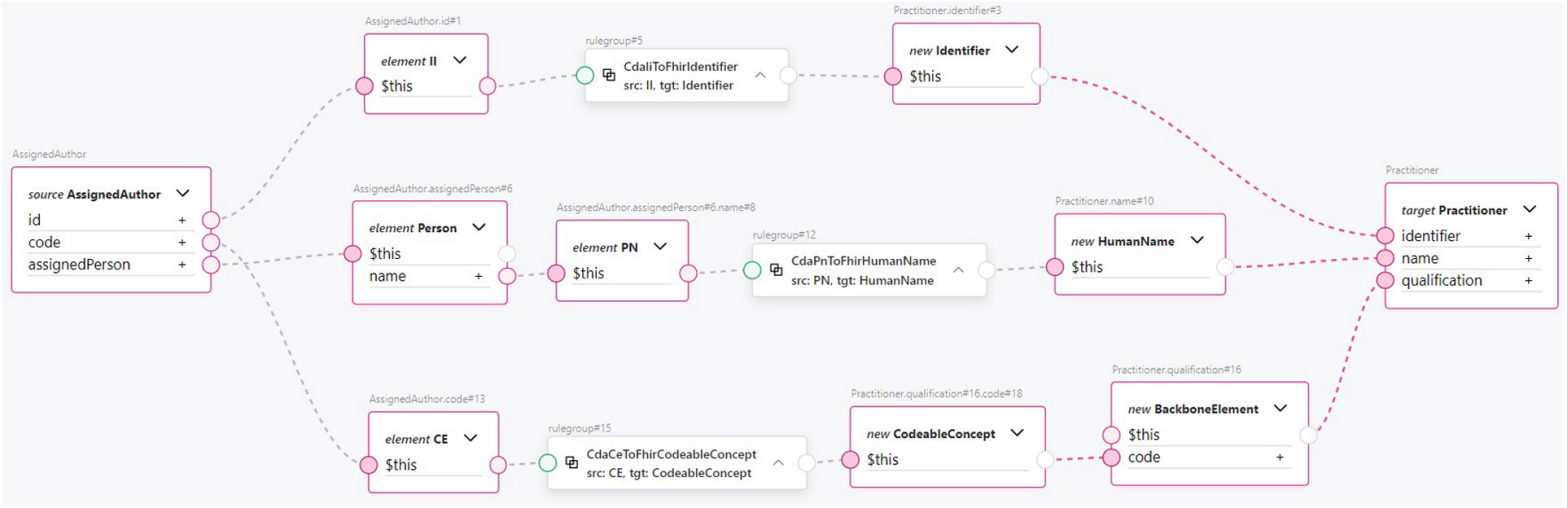
An example transformation from CDA AssignedAuthor class to FHIR Practitioner resource.

CDA class to FHIR resource transformation rules can be exemplified with Figure 9, which shows how a CDA *AssignedAuthor* class is mapped to a FHIR *Practitioner* resource using the TermX visual editor. The CDA *AssignedAuthor* class is split into the *II* data type from the id attribute, the CE data type from the code attribute, and the Person class from the *assignedPerson* attribute. Subsequently, the CDA *II* data type is transformed into the FHIR *Identifier* resource using the reusable transformation component *CdaIiToFhirIdentifier*. The CDA *CE* data type is transformed into the FHIR *CodeableConcept* resource using the reusable transformation component *CdaCeToFhirCodeableConcept*. The CDA *PN* data type is extracted from Person class and transformed into the FHIR *HumanName* data type using the reusable transformation component *CdaPnToFhirHumanName*. The transformed FHIR resources are then assigned to the target *Practitioner* resource’s identifier, qualification, and name attributes, accordingly. Notice how data type transformation rules are imported and then used. Referring to Figure 8, which shows the implementation of the *CdaCeToFhirCodeableConcept* transformation, it is clear how our approach encapsulates complexity and promotes clarity at the CDA class to FHIR resource mapping level.

#### 3.3.3 Specifying CDA section level transformations

**Figure 10:**
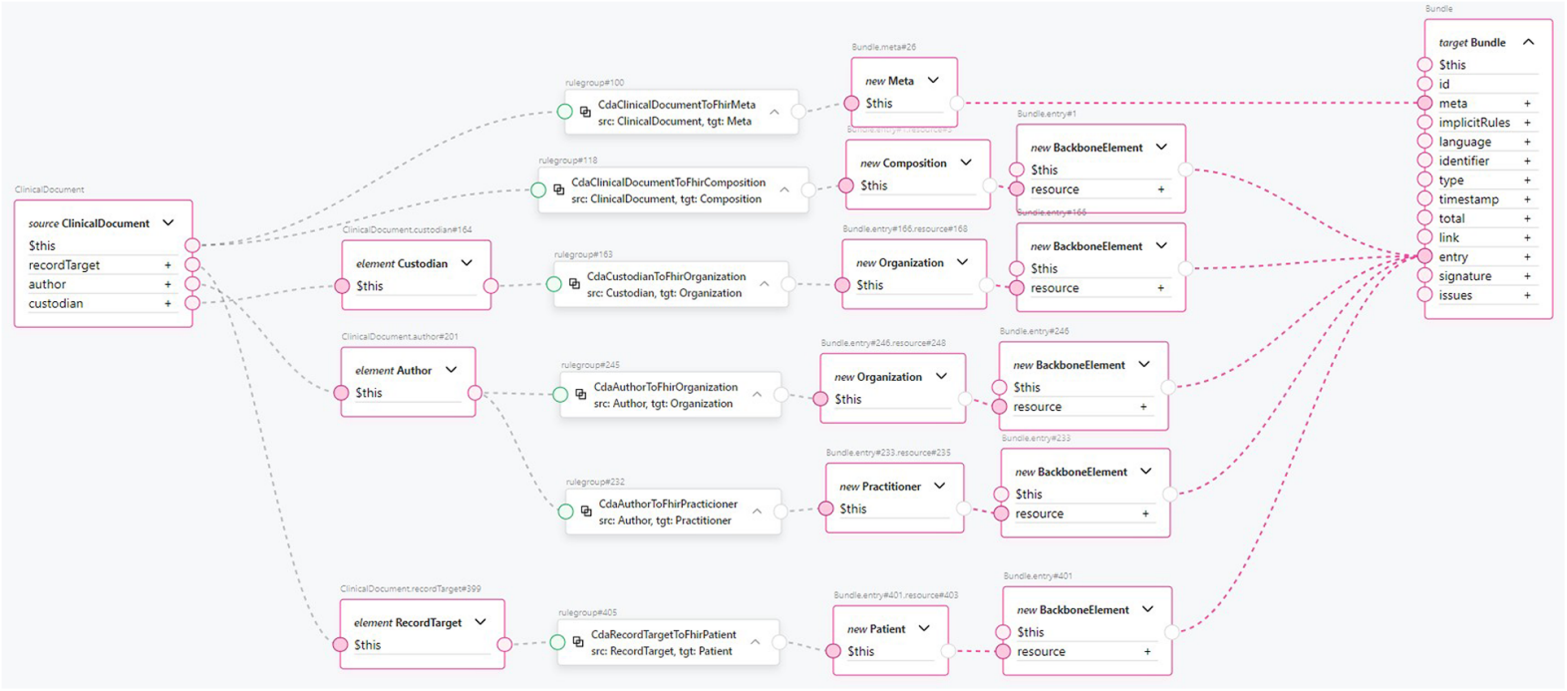
An example transformation from CDA ClinicalDocument header entries to FHIR Bundle entries.

Transforming the CDA document header to FHIR is an example of the transformation component from a CDA section to a FHIR resource. This is shown in Figure 10. The clinical document header contains a variety of information. The confidentiality codes, as top-level attributes of the header, are transformed into FHIR’s Meta resource and assigned to the FHIR Bundle’s meta attribute. The structural information about the sections in the document is compiled to form the FHIR *Composition* resource and added to the FHIR Bundle as an entry. The clinical document header’s *custodian* attribute, a CDA *Custodian* class instance, is transformed into a FHIR *Organization* resource and added to the bundle as an entry. The *author* attribute of the clinical document, a CDA *Author* class instance, contains information about the author’s person and organization. Therefore, two transformation components are used: one for transforming the data into a FHIR *Organization* resource and another for transforming the data into a FHIR *Practitioner* resource. Both resources are added to the FHIR Bundle as entries. Finally, the *recordTarget* attribute of the clinical document header, a *RecordTarget* CDA class instance, is transformed into a FHIR *Patient* resource and added to the FHIR Bundle as an entry. This concludes the scope of our *ClinicalDocument* header transformation component. The number of transformation components is approximately equal to the number of document types and CDA classes used in them, considering the CDA class hierarchy. By encapsulating transformation components such as *CdaCustodianToFhirOrganization*, *CdaAuthorToFhirOrganization*, *CdaAuthorToFhirPractitioner*, and others into reusable transformation components, the CDA header transformation rule remains comprehensible, even though the amount of information to be transformed is much larger.

#### 3.3.4 Specifying CDA document level transformations

**Figure 11:**
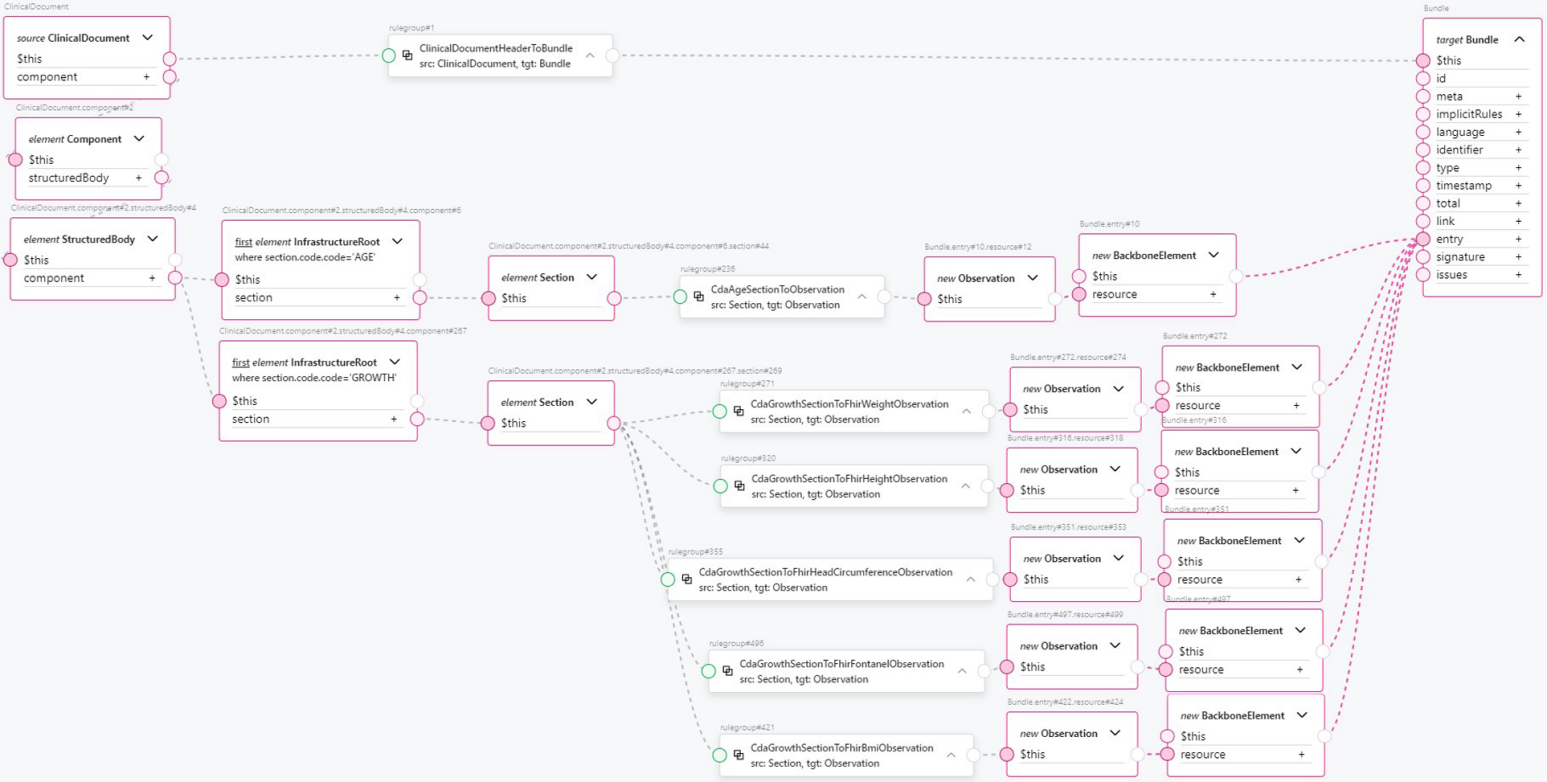
An example transformation from CDA Growth Report template to FHIR Bundle entries.

Finally, using CDA section transformation components, we compose a transformation component for the “Notice of Growth” CDA document (see Figure 11). We find a document section by section code, then apply a reusable component to transform this section into FHIR resources, and then combine them into a FHIR Bundle. The header section is extracted from the root level of the *ClinicalDocument*, while the other sections are extracted from within the <*StructuredBody*> element. From the <*structuredBody*> element, we extract two sections: the *AGE* section and the *GROWTH* section. The *AGE* section is transformed into an *Observation* FHIR resource containing the patient’s age information using a single *CdaAgeSectionToObservation* reusable transformation component. The transformed *Observation* resource is added to the FHIR Bundle as an entry. The *GROWTH* section is transformed into multiple observations, as this section contains CDA *Observation* classes in <*component*> elements for different measurements taken during the procedure: weight, height, head circumference, fontanel measurements, and body mass index. The following reusable transformation components are used:

• *CdaGrowthSectionToFhirWeightObservation*
• *CdaGrowthSectionToFhirHeightObservation*
• *CdaGrowthSectionToFhirHeadCircumferenceObservation*
• *CdaGrowthSectionToFhirFontanelObservation*
• *CdaGrowthSectionToFhirBmiObservation*

The resulting Observation FHIR resources are added to the FHIR Bundle as entries. Referring to Figure 10 for the complexity of just the CDA document header component, we see how this approach encapsulates the complexity of a single document section and enhances clarity and high-level understanding of the clinical document’s mapping to FHIR. From the data type level up to the CDA template level, the amount of code duplication is significantly reduced, as is the number of points of failure. At the same time, the clarity and comprehension of the transformations are greatly improved.

With the development of the “Notice of Growth” CDA to FHIR transformation, the following transformation components were created:

• *CdaClinicalDocumentHeaderToFhirBundle*
• *CdaAgeSectionToFhirObservation*
• *CdaGrowthSectionToFhirWeightObservation*
• *CdaGrowthSectionToFhirHeightObservation*
• *CdaGrowthSectionToFhirHeadCircumferenceObservation*
• *CdaGrowthSectionToFhirFontanelObservation*
• *CdaGrowthSectionToFhirBmiObservation*

Numerous transformation components have been created to convert CDA classes to FHIR resources and support the composition of section-level transformations. The essential components include the following:

• *CdaAssignedAuthorToFhirPractitioner*
• *CdaCustodianOrganizationToFhirOrganization*
• *CdaObservationToFhirObservation*
• *CdaOrganizationToFhirOrganization*
• *CdaPatientRoleToFhirPatient*
• *CdaEntryRelationshipToFhirObservationComponent*

The necessary data type transformation components include the following:

• *CdaAdToFhirExtendedContactDetail*
• *CdaCdToFhirCodeableConcept*
• *CdaCeToFhirCodeableConcept*
• *CdaIiToFhirIdentifier*
• *CdaIvlTsToFhirDateTime*
• *CdaPnToFhirHumanName*
• *CdaPqToFhirQuantity*
• *CdaRtoPqPqToFhirRatio*
• *CdaTelToFhirExtendedContactDetail*
• *CdaTsToFhirDate*

The *ConceptMap* *CdaAdministrativeGenderCodeToFhirGender* was also created. All these transformation components were designed to be reusable for the future development of transformation components from other CDA templates to FHIR bundles.

## 4 Analysis and discussions

### 4.1 Related work

This section provides a comprehensive review of the related work in the domain of data transformation, with a particular emphasis on the transformation process from CDA to FHIR. The related work can be systematically classified into three distinct categories: mapping languages, tools, and implementation projects. This categorization facilitates a more structured and in-depth analysis of the field.

#### 4.1.1 Mapping languages

The concept of “Mapping Language” (or Data Transformation Language) lies in establishing a platform-independent specification that can be implemented across various programming languages (70). Model-to-model transformations are typically articulated in specialized domain-specific languages, often known as model transformation languages (MTLs) (71). MTLs encapsulate algorithms that delineate the process of converting elements from one model (or multiple models) into elements of another model (or multiple models). Declarative MTLs (DTLs) only provide logic constructs to express relations between elements in these candidate models, and the execution engine is responsible for synthesizing an execution plan that uses these relations to perform the model transformation.

*Query/view/transformation*: “Query/View/Transformation” (QVT) is a specification developed by the Object Management Group (OMG) to describe transformation rules between different data models in the Model-Driven Architecture (MDA) domain (72). The language was intended to support the declarative specification of model transformations, avoid imperative constructs, and support change propagation from one model to another as well as the bi- (or multi-) directional interpretation of transformations. However, its semantics have many unclear or unsatisfactory aspects that are not precisely defined in the standard (73). The QVT Core language (QVTc) uses pattern matching as the primary logic construct. Pattern matching is done over a flat set of variables by evaluating conditions over those variables against the candidate models (74).

*eXtensible stylesheet language transformations*: XSLT is a language used to transform XML documents into other document formats or other versions of XML.^2^ XSLT is a powerful tool and a widely adopted language for transforming XML documents, including healthcare-related XML standards such as CDA. However, it is unsuitable for directly programming transformations of semantically complex models due to its low-level syntax (75). XSLT is also not a specialized language for medical data (76). One of its disadvantages is the mandatory use of XML language, which imposes limitations on use. It is also poorly readable, making it difficult to learn and debug (77).

*Whistle*: The Whistle Data Transformation Language provides a means to express mappings between schemes, enabling users to convert complex, nested data models into other equally complex and nested data formats (78). Whistle does not require a description of logical models for the data to be converted. The conversion requires only source data in JSON format and a map that describes the conversion rules. The result of the transformation is output data in JSON format.

*Liquid templates*: Liquid (79) is a templating language developed by Shopify that uses a combination of objects, tags, and filters inside template files to convert any JSON or XML format into another JSON format. A transformation engine is required to convert input data into output data based on a *.liquid template*. Microsoft FHIR Converter (80) is one such engine, processing Liquid templates to convert input data into validated FHIR format. It includes extended methods for FHIR data and is part of Microsoft’s FHIR server implementation, available in the Microsoft Azure Health Data Services product (81). Users can upload custom templates to the Azure registry, which Azure Health Data Services can then use via an API endpoint for data transformation.

*FHIR Mapping Language*: The FHIR Mapping Language (FML) (40) is a relatively new QVT-based transformation language specifically designed to transform HL7 FHIR resources to/from alternative representations, including different logical data models, FHIR resources, C-CDA documents (42), etc. (82). FML is a part of the FHIR specification. Conceptually, FML is similar to XSLT:

(1) It consists of declarative rules that are automatically matched to input data
(2) It includes a sub-language (*FHIRPath*) to reference parts of source parse trees
(3) It can reference external functions written in different languages

The source input of FML supports any object models and rendering syntaxes that conform with OMG’s Meta Object Facility (MOF)^3^ language. MOF is a general formalism for representing object models as directed acyclic graphs (DAGs). MOF-compliant models can use various syntactic constructs to represent the classes, attributes, and attribute values of such graphs. The applications of this language encompass several scenarios:

• Mapping FHIR resources across different versions of FHIR
• Converting sections of HL7 C-CDA documents into multiple FHIR resources
• Translating HL7 V2 messages into multiple FHIR resources
• Adapting any structured data format into another structured data format, including mapping to multiple FHIR resources

**Figure 12:**
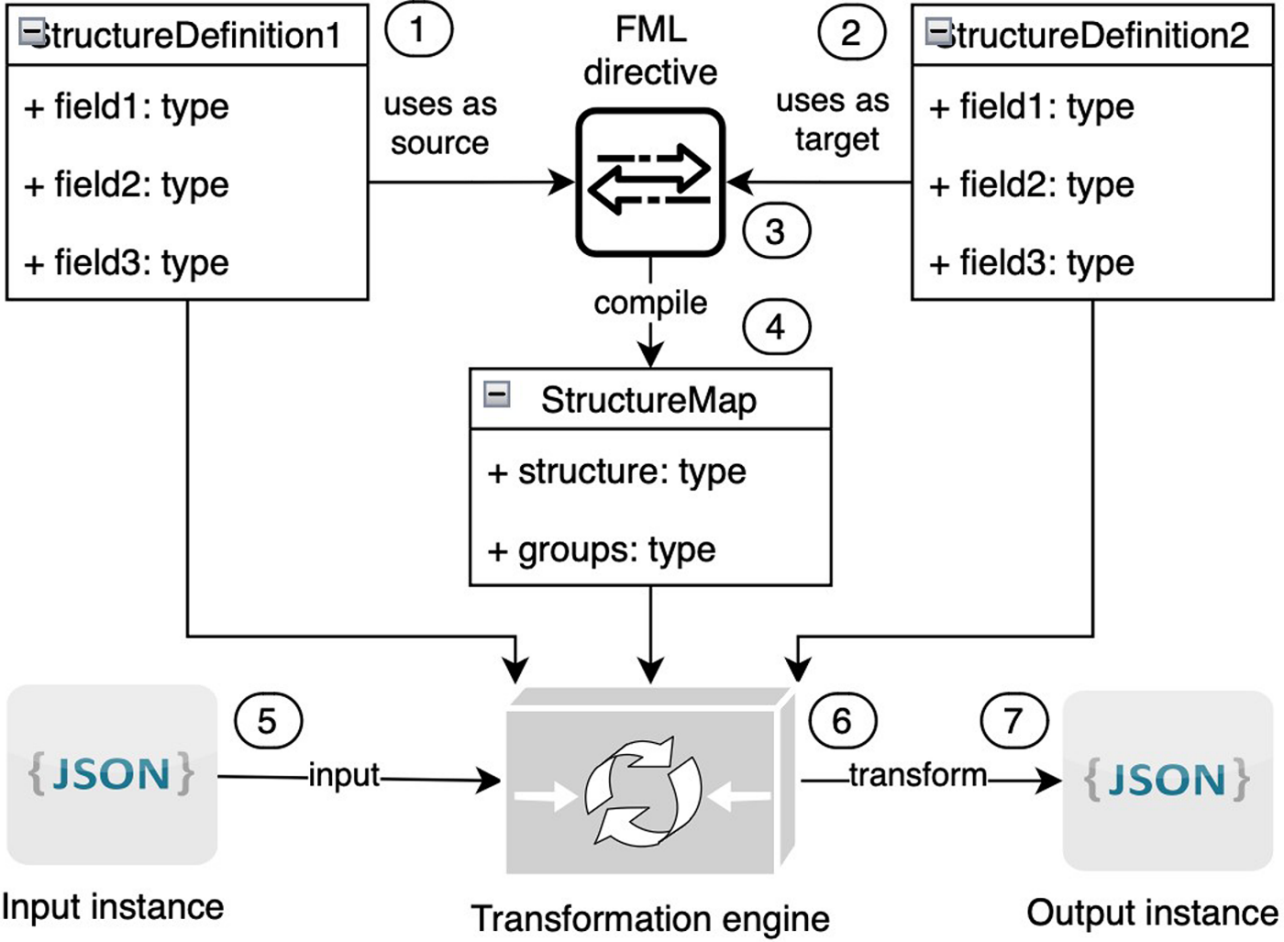
Components of FML transformation.

The technical specification of FML (40) has been published as an integral component of the FHIR specification (83). FML serves as a tool for transforming structured models from one form to another. Within the HL7 FHIR context, FML is utilized to map FHIR resources across different versions of FHIR. FML transformation requires the following (Figure 12):

• One input model (marked on the picture with the number “1”)
• At least one output model (2)
• Human-readable transformation rules (also known as FML mapping directives) (3) that outline how to transform input into output
• A machine-processable transformation map (4) created as a result of the compilation transformation rules
• One input instance that corresponds to the input model in JSON or XML format (5)
• A transformation engine (6) that will transform the input instance to the output instance (7) based on models and transformation maps

#### 4.1.2 Data transformation tools

*NextGen connect*: NextGen Connect (previously known as Mirth Connect) (84) is a robust, open-source healthcare integration engine widely used for its versatility and cost-effectiveness (85). One of its major strengths is its ability to support numerous data formats and protocols, such as HL7, XML, and JSON, making it highly adaptable to various healthcare systems (86). Its user-friendly interface and comprehensive documentation facilitate easier configuration and deployment, and the active community provides valuable support and resources. However, Mirth Connect has several drawbacks. Despite its user-friendly interface, it is primarily geared towards technical experts, making it challenging for domain experts without technical backgrounds to use it effectively (87). In our opinion, the learning curve is steep for new users unfamiliar with healthcare data standards and integration concepts. Performance can also be an issue with large-scale implementations, requiring careful optimization and resource management. Additionally, the clarity of implemented transformations can sometimes be lacking, making it difficult to understand and troubleshoot complex data flows (88). Furthermore, while the open-source version is feature-rich, some advanced features and enterprise-level support are only available in the paid version, which might limit its appeal to smaller organizations.

*Other health data integration tools*: Health data integration tools are essential for managing and transforming healthcare data, supporting interoperability within healthcare systems, and automating processes to realize cost savings. In addition to NextGen Connect, other well-known tools in this domain include Cloverleaf Integration Suite (89), Interfaceware Iguana (90), Corepoint Integration Engine (91), and Redox (92). Each tool offers numerous benefits, including connectivity and interface management, data transformation and workflow management, and support for various healthcare standards, protocols, and interfaces. They provide data mapping and support multiple data formats, leading to cost savings through reduced manual effort. However, there are challenges to consider when implementing these tools (93):

• *Complex implementation*: The process can be intricate, requiring IT professionals with expertise in healthcare data standards, protocols, and the specific tool’s configuration.
• *Initial costs*: While cost savings can be realized in the long run, initial expenses associated with software licenses, hardware, and implementation can be challenging for smaller organizations.
• *Maintenance and support*: Regular updates, troubleshooting, and addressing issues are crucial for the tool’s effectiveness, requiring dedicated resources.
• *Data mapping challenges*: Accurate and comprehensive data mapping can be challenging when dealing with disparate systems using different data standards and terminologies.
• *User training*: Staff may require training to use and navigate the tools effectively, and the learning curve can be costly.
• *Data security concerns*: Transmitting health data between systems raises data security concerns. Robust security measures are necessary to safeguard patient information and comply with data protection regulations.
• *Vendor lock-in*: Over-reliance on a specific tool or vendor can lead to potential issues if there are changes in the organization’s strategy or the vendor’s support changes.

*FML implementations*: The FHIR Mapping Language specification is implemented by code libraries such as the HAPI FHIR *StructureMap* implementation in Java (57) and its direct port to .Net (94), both of which offer transformation engines and open-source libraries. HAPI FHIR, a comprehensive Java library for FHIR, supports creating, parsing, and validating FHIR resources, providing robust tools for healthcare applications. The .Net FML implementation leverages these capabilities, bringing the same powerful functionality to the .Net ecosystem. Both libraries facilitate the transformation of healthcare data, ensuring interoperability and compliance with FHIR standards, which are crucial for modern healthcare systems.

*Matchbox*: Matchbox is an open-source initiative to support the testing and implementation of FHIR-based solutions (95). Matchbox utilizes the HAPI FHIR implementation, inheriting all its advantages while introducing additional flexibility for FML processing. Matchbox allows the preloading of FHIR implementation guides for conformance resources (*StructureMap*, *Questionnaire*, *CodeSystem*, *ValueSet*, *ConceptMap*, *NamingSystem*, *StructureDefinition*) and validates FHIR resources. Matchbox allows the defining of mapping in an FML text representation and its transformation into FHIR *StructureMap* resources. Matchbox applies the mapping to data to create FHIR-compatible data sets. Matchbox validates and executes FML transformations through the FHIR API, checking that the mapping conforms with the included validation stack.

#### 4.1.3 Implementation projects

*Austrian ELGA*: The ELGA (Elektronische Gesundheitsakte) project launched in Austria is a nationwide EHR system designed to facilitate the exchange of medical documents across healthcare providers. ELGA uses CDA to manage medical data in a document-centric format. The project supports various document types, including Physician’s Discharge Summaries, Nursing Discharge Summaries, Laboratory Reports, and Diagnostic Imaging Reports, with the addition of e-Medication reports covering prescription and medication summaries. To enhance interoperability and accessibility, recent efforts focus on mapping ELGA CDA documents to the FHIR standard using JSON mapping (96). Every element and section in JSON mapping has a “cda-path” that prescribes a rule for extracting data from a CDA document. This approach aims to generate International Patient Summaries (IPS) in FHIR format, enabling more granular access to health data and supporting cross-border healthcare data exchange within the European Union (26).

*Italian patient summary*: The Italian decree mandates that regional EHR systems support two types of documents: the Patient Summary and the Laboratory Report (27). The Patient Summary focuses on collecting the patient’s most significant clinical information and uses the CDA format. During the *eHealthNet* project, a prototype was implemented for transforming the Patient Summary from CDA to FHIR. The proposed solution included the Mapping, Extractor, and Binding components. The Mapping component contains schemas defining correspondence between an element in FHIR and another in CDA. *XPath* was used for data extraction from CDA and binding to FHIR with a series of functions written in XSLT (27).

*Swiss medications*: The Swiss healthcare system has adopted the CDA standard, incorporating specific requirements unique to Switzerland (97). This has led to the creation of the CDA-CH standards (98). Switzerland transitioned to FHIR and developed equivalent FHIR-CH specifications for medication. To verify the equivalences, mappings have been defined with the FHIR mapping language, and Matchbox has been used for transformation from CDA to FHIR and back (99). To aid this transformation process, a consolidated library of CDA templates was employed (60). The use of FML in this context facilitates the automated transformation and validation of data, ensuring compliance with FHIR profiles and enhancing the utility of Swiss health data across various healthcare scenarios.

*Estonian Andmevaatur*: The *Andmevaatur* (Data Viewer) is a tool summarizing and visualizing patient data in the ENHIS (28). The ENHIS is built upon HL7 V3 and CDA standards (100). Due to the ever-increasing volume of documents, the task of gathering observations, procedures, vaccinations, and other clinical information from documents has become increasingly time-consuming for doctors (101). *Andmevaatur* uses *xQuery* to request CDA documents from the ENHIS database, transforms them into FHIR resources using a custom-developed mapping language, and forwards the resources to the user interface application for presentation. The custom-developed mapping language includes pairs of *XPath* and *FHIRPath* and a Java adapter for their execution. *XPath* is used for data extraction from CDA and *FHIRPath* is used for inserting data into the appropriate place in the FHIR resource. The development of an independent mapping language has been discontinued, and migration to FML is planned. Using *Andmevaatur*, doctors can save at least three minutes per visit, which is approximately 15 percent of the time typically spent interacting with a patient (101).

### 4.2 Comparison of languages, tools, and implementations

To find the most suitable tool for our needs, we embarked on a comprehensive comparison of various languages, implementations, and tools. Our evaluation was based on a set of carefully developed criteria; the results are summarized in Table 2 and the conclusion is as follows:

• *Strict data model support*: DTL-based languages, such as FML, and their implementations provided robust support for strict data models.
• *Reuse of transformation*: We found that all languages used in evolution, along with their implementations and software, commendably support the reuse of transformations.
• *FHIR native support*: FML implementations, Matchbox, and TermX may be classified as tools with native FHIR support.
• *Executable software*: All implementations and software are classified as executable software.
• *Open-source license*: All languages, implementations, and software, except for NextGen Connect, and tools in the section “Other health data integration tools” are available under open-source licenses, promoting transparency and collaboration.
• *Visual transformation editor*: TermX and the health data integration tools stood out with their visual editors, which greatly facilitate the management of transformation flow.

**Table 2:** Evaluation of artifacts

Artifact	Strict data models	Reuse	Native FHIR support	Execu- table software	Open-source	Visual editor
Query/View/Transformation (QVT) language (4.1.1)	+	+	−	−	+	−
Extensible Stylesheet Language Transformations (XSLT) (4.1.1)	+	+	−	−	+	−
Whistle (4.1.1)	−	+	−	+	+	−
Liquid (4.1.1)	−	+	−	+	+	−
FHIR Mapping Language (FML) (4.1.1)	+	+	+	−	+	−
FML implementations (4.1.2)	+	+	+	+	+	−
Integration tools (4.1.2)	+/−	+	−	+	−	+/−
Matchbox (4.1.2)	+	+	+	+	+	−
TermX (2.2)	+	+	+	+	+	+

Notes: “+” indicates that the criterion is met, while “−” indicates that it is not met.

After a comprehensive evaluation, it became evident that none of the existing implementations or tools were suitable, as they did not meet all of our selection criteria. This aligns with the health data interoperability issues highlighted in various recent papers by other implementers (27, 96).

In response to this, we developed the TermX FML Editor using the DS methodology. The designers behind TermX leveraged the existing FML language and the HAPI FHIR implementation, validating and reusing them to mitigate the risk of failure. Upon evaluating TermX, it was unequivocally clear that it was the only solution that met all of our selection criteria, thereby establishing it as the optimal choice for our needs.

### 4.3 Evaluation of visual reusable transformation rules

#### 4.3.1 Toward federated interoperability in the EHDS

Ensuring federated interoperability (23, 24) is essential in the EHDS as it reduces administrative, operational, and international coordination costs. Federated systems store data in appropriate locations and formats, avoiding the complexity of large central repositories (102). This respects data sovereignty and privacy rules while allowing interoperability and independent innovation (103).

Centralized systems require significant infrastructure investment and management, which can be inefficient. Federated systems distribute these responsibilities, leveraging existing infrastructure and expertise and reducing compliance burdens with diverse regulatory frameworks. Federated semantic interoperability facilitates real-time data sharing, which is crucial for informed healthcare decision-making. By enabling seamless health data exchange, federated systems support innovative healthcare solutions, such as integrated care platforms and personalized medicine networks, enhancing care quality and patient outcomes.

Federated interoperability also supports EHDS initiative evaluations by providing a robust data integration and analysis framework, essential for assessing health interventions and informing policy decisions. Leveraging diverse data sources without extensive migration accelerates innovation and evaluation in healthcare. However, an effective system for semantic data transformation is required, as subsystems use different standards and models. The EHDS will inevitably need semantic data transformation, necessitating the evolution of user-friendly tools such as TermX.

#### 4.3.2 Empowering domain experts

Achieving semantic interoperability is challenging due to the complexity of data transformation processes, which traditionally require significant technical expertise. The proposed techniques and TermX tool enable domain experts with minimal technical skills to participate effectively. The visual editor allows them to create and manage data transformation rules through an intuitive interface, democratizing the process and reducing reliance on technical specialists. This expedites development and deployment, improving the efficiency and scalability of interoperability initiatives.

The TermX tool explained in this paper allows domain experts to develop and validate data transformation rules, accommodating the evolving landscape of health standards and technologies (104). Direct involvement of domain experts ensures accuracy and relevance, as they bring a deep understanding of specific data and context. This collaboration fosters a more comprehensive approach to data transformation, enhancing the quality and reliability of interoperable data. The tool’s validation features enable domain experts to test and refine transformation components, ensuring that transformed data meets expected standards and requirements and contributes to effective and trustworthy interoperability solutions.

#### 4.3.3 Continuous adaptation to emerging innovations

Achieving federated semantic health data interoperability is crucial for supporting innovation within the EHDS (17). The healthcare data landscape constantly evolves, driven by innovations and new requirements. Semantic interoperability requires continuous adaptation. The proposed techniques and TermX tool support a flexible, modular approach to data transformation, adapting to new standards and technologies as they emerge. This ensures long-term interoperability and prevents obsolescence.

For instance, the transition from CDA to FHIR represents a significant shift in data structuring and exchange. As new versions of these standards are released, the tool must incorporate these changes, facilitating seamless data transformation. This capability allows healthcare organizations to leverage the latest advancements without significant disruptions or reengineering.

The evolving standards highlight the need for a collaborative approach to interoperability. The tool leverages collective expertise to stay updated with the latest developments by fostering a community-driven repository of transformation components and best practices. This promotes continuous improvement and innovation in health data interoperability.

#### 4.3.4 Open FAIR access to routine clinical data

The FAIR (Findable, Accessible, Interoperable, Reusable) data principles are key enablers of secondary data use for societal benefit (105). Opening FAIR access to routine clinical data can drive advancements in medical research, clinical trials, public health, and policy-making (2–4, 106). Achieving FAIR access while maintaining privacy and security is challenging and requires robust technical solutions (18). Federated semantic interoperability offers a solution by keeping data in its original location, ensuring privacy, and enabling the integration and analysis of anonymized or pseudonymized data.

The proposed techniques and TermX tool support FAIR principles by providing a framework for transforming and integrating clinical data in a standardized manner. This ensures that data is findable and accessible, consistently represented, and understood. By facilitating data reuse through interoperable transformation rules, the tool enhances the utility of clinical data for secondary purposes. Leveraging routine clinical data for secondary use has profound societal implications, providing researchers with data for studies, enabling public health officials to monitor and respond to health threats, and guiding policymakers with evidence-based insight (107).

#### 4.3.5 Integrating health data with other sectors

Health data is interconnected with data from sectors such as education, social services, the environment, and the economy (108, 109). Integrating health data with these sectors is essential for a holistic understanding of health determinants and outcomes, as the World Health Organization (WHO) recommends (110).

Although TermX was designed with FHIR support for health data interoperability, it is versatile enough to integrate and facilitate interoperability with other data sets beyond healthcare. This adaptability allows TermX to connect health data with various sectors, such as education, social services, the environment, and the economy. TermX supports a more comprehensive analysis of factors influencing health outcomes by enabling seamless data exchange across these domains. This flexibility ensures that TermX can serve as a powerful tool for creating holistic data ecosystems where health data is enriched by insights from other sectors, ultimately contributing to more informed decision-making and improved public health strategies.

#### 4.3.6 Toward resolving three health data dilemmas

Klementi et al. (18) identified three health data dilemmas: accessibility, comprehensiveness, and ownership. The *accessibility dilemma* involves balancing health data access for improved outcomes with protecting sensitive information. Ensuring FAIR (Findable, Accessible, Interoperable, and Reusable) access often conflicts with data protection requirements (111–113). The *comprehensiveness dilemma* concerns creating a complete health record from fragmented data stored across various systems. Issues such as semantic interoperability and legal barriers impede the consolidation of data into a comprehensive personal health record (PHR) (114). The *ownership dilemma* addresses the conflict between individuals’ rights to control their health data and the practical difficulties of exercising these rights (115, 116).

An EHDS architecture where individuals own and control their health data could use decentralized content-addressable storage networks (18). The proposed techniques and TermX tool create conditions that enable individuals to share their health data with healthcare professionals and ensure FAIR access to routine clinical data for secondary use (117, 118). This empowers more stakeholders to participate in the data transformation process, keeping health data interoperability at the forefront of healthcare innovation.

### 4.4 Implementation scenarios

#### 4.4.1 Execution of the transformations in the single installation

The technical implementation of the solution encompasses both the design and transformation phases. This paper focuses on the design phase, wherein data models and transformations are developed. The resulting artifacts can be stored either in GitHub or on a FHIR server. The TermX Editor is utilized for the design and testing of these transformations, but it is not required for their execution. For execution purposes, libraries such as HAPI FHIR, .Net, or their equivalents can be employed to compile and run the transformations. To enhance throughput, the application should support the caching of the utilized models (StructureDefinition instances) and compiled transformations (StructureMap instances). This application can function as a standalone service or as a module integrated into the FHIR server.

#### 4.4.2 The transformations in the context of EHDS

**Figure 13:**
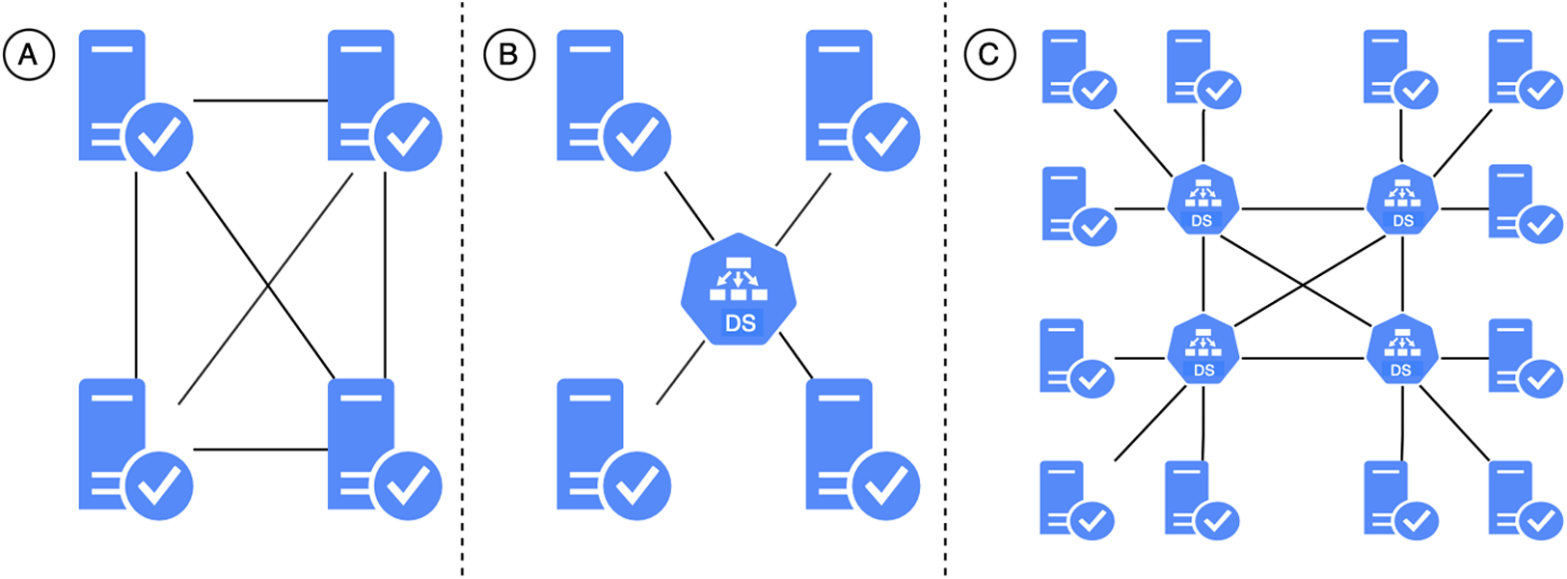
Possible topologies of EHDS: **(A)** every node is connected to every other node, **(B)** there is a central node to which all other nodes are connected, **(C)** a hybrid of topologies.

When integrating two systems, two data models (source and target) and one set of transformations are required for one-way transformations or two sets for bidirectional transformations. If we consider that each medical system in the EHDS integrates with every other system and each has a unique data model, there will be N data models, resulting in an integration network with a complexity of O(2^n^) (Figure 13A). By creating a central model, we would have N+1 models and N (for one-way) or N*2 (for bidirectional) sets of transformations (Figure 13B). However, a single central model for all European countries is not realistic (9). It would be beneficial to reduce the number of models by creating smaller Data Spaces, where institutions within a country or region share a single model. Instead of a single central model, domain-specific Data Spaces could be established, connecting all EU laboratories (119), immunization records (120, 121), or radiology services into unified networks (Figure 13C). Such grouping would reduce the number of transformations and administrative burdens.

### 4.5 Limitations

#### 4.5.1 Use-case-specific mapping of components

The current study was conducted and validated for a specific use case, namely the transformation of ENHIS documents. When comparing documents from Estonia with those from other countries, we find that documents of the same type, such as outpatient summaries, differ in the number of sections, section labeling, and terminology used. Additionally, country-specific extensions may be used. This implies that for each specific implementation, the representation at the business domain knowledge level may differ, and the set of transformations developed in this research study may require adaptation.

The foundational resources from the CDA and FHIR frameworks are highly compatible and could be suitable for use in any country. The ISO 23903 Interoperability and Integration Reference Architecture addresses the challenges associated with integrating such models and frameworks. Examples include mappings of HL7 V2 and HL7 V3 models and specifications, and the re-engineering and mapping of the higher-level specifications ISO 12967 Health Informatics Service Architecture and ISO 13940:2015 System of concepts to support continuity of care (122).

Although the detailing of base types in mapping may vary depending on the use case, for ENHIS, mapping of the CDA II to FHIR Identifier data types requires only the transformation of key attributes “root” to “system” and “extension” to “value” (Figure 4). However, in another information system, additional attributes such as “display” and “use” might be required, which we have not mapped, as this mapping is specific to the given use case. Nevertheless, it is easily generalizable if we extend the use case.

#### 4.5.2 Mapping correctness

Actors from different scientific domains and disciplines, different communities, and different policy domains represent and understand related concepts differently (123). This decision on correct mapping is only possible at the business domain knowledge level, represented through domain ontologies and related terminologies.

• *Validation by analyst*. Business analysts, as domain experts, possess comprehensive knowledge of the domain’s ontology and terminology. They are responsible for planning and ensuring the accuracy of transformations. TermX is a robust tool specifically designed for analysts. Consequently, business analysts are well-equipped to make transformation decisions and verify the accuracy of transformations by manually performing a reasonable number of tests.
• *Technical validation*. The technical validation of transformation correctness can be achieved through various methodologies. Section 2.3.4 elaborates on validation utilizing Natural Language Processing (NLP). Nevertheless, the ISO 23903 Interoperability and Integration Reference Architecture facilitates the accurate mapping of components across business, informational, computational, and engineering viewpoints. This framework supports the design and management of systems across diverse domains and contexts, thereby ensuring interoperability among ecosystem components (124).

Technical validation of transformations will make up future work.

## 5 Conclusion

Transforming health data from CDA to FHIR format is critical to achieving health data semantic interoperability. This paper presents generalized techniques for utilizing the TermX tool to develop reusable data transformation components and verify that the designed transformation components accurately transform data as expected. TermX leverages the FHIR Mapping Language to facilitate complex and technical data transformations. It is designed explicitly for domain experts, enabling them to develop and manage data transformation rules with minimal technical knowledge.

The pressing need for such a tool arises from the ongoing evolution of the ENHIS, which is transitioning EHRs from CDA to FHIR (22). This transition is not only a technical upgrade but also a strategic move to enhance health data’s flexible and on-time semantic interoperability to improve the quality of clinical care and control healthcare costs, ensuring that patients’ health information can be seamlessly shared and understood across systems and by healthcare practitioners in real time. Since vast amounts of historical EHR data in the ENHIS are stored in various HL7 CDA formats (15), transforming this data dynamically to FHIR as needed, rather than permanently, is essential. This approach utilizes federated semantic health data interoperability, ensuring that historical EHR data remains immutable but interoperable and accessible without requiring extensive and costly data migration efforts from one data repository and format to another.

The TermX tool was developed using the Design Science (DS) methodology, which emphasizes the creation and evaluation of artifacts designed to solve the problems identified. In the problem investigation phase, we conducted an analysis of languages, implementations, and tools to find a possible solution and tool to meet the ENHIS data transformation requirements. As we found no suitable solution or tool, and because the same health data interoperability issues were stressed in various recent papers, we developed TermX using the DS approach. TermX was designed (treatment design phase of DS) through the generalization, abstraction, and formalization of the needs of the ENHIS, ensuring that it is universal, usable, practical, and effective in most real-world health data transformation applications. The tool provides a visual editor for developing transformation components with FHIR Mapping Language support for transforming data from any data structure to any other. We evaluated (treatment validation phase of DS) that this tool might be usable and valuable for domain experts who may not have deep technical knowledge of information and communication technology. In the treatment implementation phase (not part of the DS but of the engineering cycle), we implemented the TermX solution with funding from the Estonian Business and Innovation Agency.

### 5.1 Research contribution

The primary business need addressed by the TermX tool is the efficient and validated transformation of health data from one data format to another. As healthcare organizations increasingly move toward adopting the FHIR standard, such tools are critical to bridge the semantic interoperability issues related to the concurrent utilization of legacy and new health data formats. Enabling domain experts to create and manage formal data transformation components in a simple WYSIWYG way using a visual editor, TermX reduces the need for technical specialists, which ultimately reduces costs and speeds up the deployment process needed to transform health data. Moreover, TermX ensures that data transformations can be carried out on the fly according to federated semantic interoperability, allowing data to be stored in different data formats while ensuring that healthcare providers have continuous and uniform access to both old and new data, in turn ensuring continuity of care and clinical decisions.

Socially, the implications of enhanced semantic interoperability are profound. Improved data interoperability means healthcare providers can share information more effectively, leading to better care coordination, reduced medical errors, and improved patient outcomes. This translates into more timely and accurate diagnoses, personalized treatment plans, and ultimately better patient health outcomes. Furthermore, integrating and analyzing data from diverse sources supports public health initiatives, research, and policy-making, contributing to the overall improvement of healthcare systems. The evaluation of the TermX tool demonstrated its effectiveness in developing reusable transformation components that domain experts can use for health data transformations. The tool was tested to ensure that the transformations were accurate and that they met the expected standards. The results showed that TermX could reliably perform the necessary transformations, supporting the hypothesis that a visual editor for the FHIR mapping language is both feasible and beneficial.

### 5.2 Future research and evaluation directions

While the TermX tool has shown promise, there are several areas for future research and development. One key area is the continuous improvement of the tool’s user interface and experience, ensuring that it remains intuitive and accessible for domain experts. Additionally, expanding the tool’s capabilities to handle more complex transformation scenarios and integrating machine learning techniques to suggest optimal transformation rules could further enhance its utility. Another important direction is developing a comprehensive evaluation framework to continuously assess the quality and performance of the transformations. This framework could include metrics for measuring the accuracy, completeness, efficiency, user satisfaction, and adoption rates of transformations. Finally, fostering collaboration and knowledge-sharing among users of the TermX tool could lead to the development of a community-driven repository of transformation components and best practices. This repository could be a valuable resource for healthcare organizations worldwide, facilitating the broader adoption of FHIR and realizing truly interoperable health information systems.

### 5.3 Conclusion summary

In conclusion, the TermX tool represents a significant advancement in the quest for the unified federated semantic interoperability of health data. The tool addresses critical business and social needs by enabling domain experts to develop and manage transformation components with FHIR Mapping Language support. It supports the efficient and accurate transformation of health data, ensuring that historical data remains accessible and interoperable. As healthcare systems continue to evolve, tools such as TermX will play a crucial role in ensuring that data interoperability remains at the forefront of these advancements, ultimately leading to improved healthcare outcomes for patients and more efficient healthcare systems.

By addressing these critical areas, the TermX tool not only meets the immediate needs of the Estonian National Health Information System but also sets a precedent for other health systems seeking to enhance their data interoperability capabilities.

**What was known on the topic:**

(1) The EHDS aims to construct a health data-sharing ecosystem within the European Union, establishing rules and common standards to facilitate the use of EHRs.
(2) Each country that uses CDA tackles the transformation from CDA to FHIR in its own unique way, suggesting that there is no one-size-fits-all solution.
(3) Previously, no tools were available in the healthcare field for visualizing transformation with FHIR support.

**What this study added to our knowledge:**

(1) In the federated approach, systems that join the EHDS can store data in a location and format that suits them and transform the data to the EHDS standard in real time.
(2) TermX provides the ability to define and manage transformation components in a visual editor using the FML Mapping Language and strict data structures, such as FHIR resources and CDA classes.
(3) TermX enhances clarity, enables the reuse of transformation components, conceals the complexity of the FML mapping language, and allows analysts to quickly adapt to its usage.

## Data Availability

The TermX project^4^ is available on GitHub, including the source code of TermX modules and applied projects. TermX modules include server, web application, and FML Editor (38). The source code of the developed CDA to FHIR transformations and the related presentations and screenshots are published in the TermX “cda2fhir” repository^5^.

## Footnotes

^1^What you see is what you get.

^2^https://www.w3.org/TR/xslt-30.

^3^http://www.omg.org/mof/.
